# Growth disrupting mutations in epigenetic regulatory molecules are associated with abnormalities of epigenetic aging

**DOI:** 10.1101/gr.243584.118

**Published:** 2019-07

**Authors:** Aaron R. Jeffries, Reza Maroofian, Claire G. Salter, Barry A. Chioza, Harold E. Cross, Michael A. Patton, Emma Dempster, I. Karen Temple, Deborah J.G. Mackay, Faisal I. Rezwan, Lise Aksglaede, Diana Baralle, Tabib Dabir, Matthew F. Hunter, Arveen Kamath, Ajith Kumar, Ruth Newbury-Ecob, Angelo Selicorni, Amanda Springer, Lionel Van Maldergem, Vinod Varghese, Naomi Yachelevich, Katrina Tatton-Brown, Jonathan Mill, Andrew H. Crosby, Emma L. Baple

**Affiliations:** 1 Institute of Biomedical and Clinical Science, University of Exeter Medical School, RILD Wellcome Wolfson Centre, Royal Devon and Exeter NHS Foundation Trust, Exeter, EX2 5DW, United Kingdom;; 2 Genetics Research Centre, Molecular and Clinical Sciences Institute, St. George's University of London, London SW17 0RE, United Kingdom;; 3 Human Genetics and Genomic Medicine, Faculty of Medicine, University of Southampton, Southampton, SO16 6YD, United Kingdom;; 4 Wessex Clinical Genetics Service, Princess Anne Hospital, Southampton, SO16 5YA, United Kingdom;; 5 Department of Ophthalmology and Vision Science, University of Arizona School of Medicine, Tucson, Arizona 85711, USA;; 6 Department of Clinical Genetics, Copenhagen University Hospital, Blegdamsvej 3B, 2200 Copenhagen N, Denmark;; 7 Northern Ireland Regional Genetics Centre, Clinical Genetics Service, Belfast City Hospital, Belfast, BT9 7AB, United Kingdom;; 8 Monash Genetics, Monash Health, Clayton, Victoria, VIC 3168, Australia;; 9 Institute of Medical Genetics, University Hospital of Wales, Cardiff, CF14 4XN, United Kingdom;; 10 North East Thames Regional Genetics Service and Department of Clinical Genetics, Great Ormond Street Hospital, London, WC1N 3JH, United Kingdom;; 11 University Hospitals Bristol, Department of Clinical Genetics, St Michael's Hospital, Bristol, BS2 8EG, United Kingdom;; 12 UOC Pediatria ASST Lariana, Como, Italy;; 13 Department of Paediatrics, Monash University, Clayton, Victoria, VIC 3168, Australia;; 14 Centre de génétique humaine and Clinical Investigation Center 1431 (INSERM), Université de Franche-Comté, 25000, Besançon, France;; 15 Clinical Genetics Services, New York University Hospitals Center, New York University, New York, New York 10016, USA;; 16 Division of Genetics and Epidemiology, Institute of Cancer Research, London SM2 5NG, United Kingdom;; 17 South West Thames Regional Genetics Service, St. George's University Hospitals NHS Foundation Trust, London SW17 0QT, United Kingdom;; 18 Peninsula Clinical Genetics Service, Royal Devon and Exeter Hospital, Exeter, EX1 2ED, United Kingdom

## Abstract

Germline mutations in fundamental epigenetic regulatory molecules including DNA methyltransferase 3 alpha (*DNMT3A*) are commonly associated with growth disorders, whereas somatic mutations are often associated with malignancy. We profiled genome-wide DNA methylation patterns in *DNMT3A* c.2312G > A; p.(Arg771Gln) carriers in a large Amish sibship with Tatton-Brown–Rahman syndrome (TBRS), their mosaic father, and 15 TBRS patients with distinct pathogenic de novo *DNMT3A* variants. This defined widespread DNA hypomethylation at specific genomic sites enriched at locations annotated as genes involved in morphogenesis, development, differentiation, and malignancy predisposition pathways. TBRS patients also displayed highly accelerated DNA methylation aging. These findings were most marked in a carrier of the AML-associated driver mutation p.Arg882Cys. Our studies additionally defined phenotype-related accelerated and decelerated epigenetic aging in two histone methyltransferase disorders: *NSD1* Sotos syndrome overgrowth disorder and *KMT2D* Kabuki syndrome growth impairment. Together, our findings provide fundamental new insights into aberrant epigenetic mechanisms, the role of epigenetic machinery maintenance, and determinants of biological aging in these growth disorders.

DNA methylation is an essential epigenetic process involving the addition of a methyl group to cytosine. It is known to play a role in many important genomic regulatory processes, including X-Chromosome inactivation, genomic imprinting, and the repression of tumor suppressor genes in cancer, mediating transcriptional regulation as well as genomic stability (Jones 2012). Three catalytically active DNA methyltransferases (DNMTs) are involved in the methylation of cytosine: DNMT1, which is mainly responsible for the maintenance of DNA methylation over replication, and DNMT3A and DNMT3B, which generally perform de novo methylation of either unmethylated or hemimethylated DNA. An absence of these enzymes in mice results in embryonic (DNMT1 and 3B) or postnatal (DNMT3A) lethality (Okano et al. 1999), confirming their essential roles in development. In line with knockout mouse models, pathogenic variants affecting the chromatin binding domains of DNMT1 have been shown to cause two separate progressive autosomal dominant adult-onset neurologic disorders (Klein et al. 2011). Biallelic pathogenic variants in *DNMT3B* have been associated with immunodeficiency, centromere instability, and facial anomalies (ICF) syndrome (Jiang et al. 2005). To date, DNMT3A has been linked to a number of physiological functions, including cellular differentiation, malignant disease, cardiac disease, learning, and memory formation. Somatically acquired pathogenic variants in *DNMT3A* are associated with >20% of acute myeloid leukemia (AML) cases, whereas heterozygous germline pathogenic loss-of-function variants have been found to underlie Tatton-Brown–Rahman syndrome (TBRS; also known as DNMT3A-overgrowth syndrome, OMIM 615879) (Challen et al. 2011; Tatton-Brown et al. 2014). TBRS is characterized by increased growth, intellectual disability (ID), and dysmorphic facial features. More recently, heterozygous gain-of-function *DNMT3A* missense variants affecting the DNMT3A PWWP domain have been shown to cause microcephalic dwarfism and hypermethylation of Polycomb-regulated regions (Heyn et al. 2019).

There is an emerging group of epigenetic regulatory molecule-associated human growth disorders in which the underlying molecular defect is a disruption to the DNA methylation and histone machinery. There are now over 40 disorders identified within this group, which can be further subgrouped into diseases resulting from disruption of the “writers,” “readers,” and “erasers” of epigenetic modifications (Bjornsson 2015). Example disorders in each group include Kabuki, Sotos, and Weaver syndromes (“writers”); Smith-Magenis, Rett, and Bohring–Opitz syndromes (“readers”); and Wilson–Turner and Cleas–Jensen syndromes (“erasers”). The final subgroup occurs because of disruption of chromatin remodelers, with example resulting disorders including CHARGE and Floating–Harbor syndromes. Neurological and cognitive impairment are common features of these conditions, suggesting that precise epigenetic regulation may be critical for neuronal homeostasis. However, a true understanding of the pathogenic mechanism underlying these conditions remains poorly understood.

In the current study, we investigated the methylomic consequences of a *DNMT3A* pathogenic variant (NC_000002.12:g.25240312C > T; NM_022552.4:c.2312G > A; p.(Arg771Gln)) in a large Amish family comprising four individuals affected with TBRS arising as a result of a mosaic pathogenic *DNMT3A* variant in their father (Xin et al. 2017). The occurrence of multiple affected and unaffected individuals in the same sibship, together with the combined genetic and environmental homogeneity of the Amish, permitted an in-depth investigation of the genome-wide patterns of DNA methylation associated with pathogenic variation in *DNMT3A*. We subsequently extended our analyses to other (non-Amish) TBRS patients harboring distinct pathogenic de novo *DNMT3A* variants, as well other methyltransferase-associated overgrowth and growth deficiency syndromes, defining altered epigenetic profiles as common key themes of these growth disorders.

## Results

### Reduced DNA methylation at key sites involved in morphogenesis, development, and differentiation in TBRS patients

**Figure 1.**
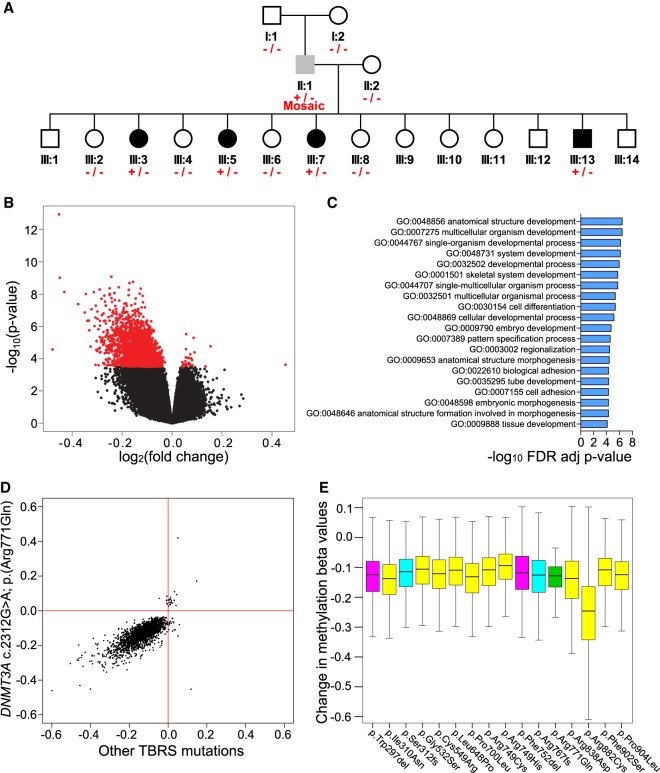
TBRS *DNMT3A* variants are associated with widespread DNA hypomethylation. (*A*) Simplified pedigree indicating the genotyping of individuals in the Amish family investigated: (+/−) heterozygous carriers of the *DNMT3A* c.2312G > A p.(Arg771Gln) variant; (+/− Mosaic) the *DNMT3A* c.2312G > A p.(Arg771Gln) mosaic father; (−/−) wild-type individuals. Black shading indicates individuals with a phenotype consistent with TBRS, gray shading, the father with macrocephaly and mild intellectual impairment; and white shading, unaffected individuals. Each of these samples was profiled on the Illumina 450K DNA methylation array. (*B*) Volcano plot showing site-specific DNA methylation differences (*x*-axis) and −log_10_ *P*-values (*y*-axis) from an analysis comparing Amish *DNMT3A* c.2312G > A; p.(Arg771Gln) pathogenic variant carriers and wild-type family members using the Illumina 450K array. Red values indicate the 2606 differentially methylated positions (DMPs) detected at a Benjamini–Hochberg FDR < 0.05. (*C*) Top 20 Gene Ontology enrichment analysis categories associated with the 2606 DMPs identified in *DNMT3A* c.2312G > A; p.(Arg771Gln) pathogenic variant carriers versus wild-type family members. (*D*) Comparison of *DNMT3A* c.2312G > A; p.(Arg771Gln) identified DMPs (log_2_ fold change) relative to other *DNMT3A* TBRS-associated variants assessed in this study (all variants grouped and measured relative to controls). Pearson correlation coefficient = 0.6620, *P*-value <2.2 × 10^−16^. (*E*) Boxplot illustrating the DNA methylation changes observed in association with the *DNMT3A* TBRS variants studied at the DMPs identified in the Amish *DNMT3A* c.2312G > A p.(Arg771Gln) carriers. The predicted protein consequence of each *DNMT3A* variant studied is indicated: Pink indicates in-frame deletion; yellow, single-nucleotide variant; cyan, duplications predicted to result in a frameshift; green, Amish c.2312G > A; p.(Arg771Gln) variant.

*DNMT3A* encodes a DNMT with both de novo and maintenance activity (Okano et al. 1999; Chen et al. 2003). We first looked for global changes in DNA methylation in whole blood obtained from *DNMT3A* c.2312G > A; p.(Arg771Gln) carriers, using the methylation-sensitive restriction enzyme–based luminometric methylation assay (LUMA) (Karimi et al. 2006) to quantify DNA methylation across GC-rich regions of the genome, finding no evidence for altered global DNA methylation (LUMA: mean *DNMT3A* c.2312G > A carriers = 0.274, wild type = 0.256, *t*-test *P*-value = 0.728). We next quantified DNA methylation at 414,172 autosomal sites across the genome using the Illumina 450K array. Globally, a subtle decrease in mean DNA methylation was noted in available age/sex-matched *DNMT3A* heterozygous c.2312G > A; p.(Arg771Gln) individuals compared with their matched unaffected sibling samples, although this was not statistically significant (Wilcoxon rank-sum test *P*-values for two matched pairs = 0.24 and 0.14) (Supplemental Fig. S1). In contrast, an analysis of site-specific DNA methylation differences in *DNMT3A* c.2312G > A; p.(Arg771Gln) carriers (including the mosaic father) versus wild-type individuals in the Amish pedigree identified 2606 differentially methylated positions (DMPs; Benjamini–Hochberg false discovery rate [FDR] < 0.05) (Fig. 1A,B; Supplemental Table S1), of which 1776 DMPs were characterized by a >10% change in DNA methylation. Supplemental Figure S2 also highlights DNA methylation levels at these DMPs across all carriers and control individuals profiled in this study. Technical validation of Illumina 450K array data was performed using bisulfite pyrosequencing for three top-ranking DMPs, confirming significant differences in *DNMT3A* c.2312G > A; p.(Arg771Gln) carriers at each of the tested loci (Supplemental Fig. S3).

The DMPs identified were highly enriched for sites characterized by reduced DNA methylation in *DNMT3A* c.2312G > A; p.(Arg771Gln) heterozygotes (*n* = 2576 DMPs, 98.85%, sign-test *P*-value <2.2 × 10^−16^). Although there were no statistically significant differences between DNA methylation-based blood cell composition estimates derived from our data (Supplemental Table S2), we examined the extent to which the identified DMPs were potentially influenced by cell-type differences between *DNMT3A* c.2312G > A; p.(Arg771Gln) carriers and wild-type family members. There was a highly significant correlation (*r* = 0.876, *P*-value <2.2 × 10^−16^) (Supplemental Fig. S4) in effect sizes at the 2606 DMPs between models, including and excluding cell types as covariates, indicating that the observed patterns of differential DNA methylation are not strongly influenced by cell-type variation. We used DMRcate (Peters et al. 2015) to identify spatially correlated regions of differential DNA methylation significantly associated with the *DNMT3A* c.2312G > A; p.(Arg771Gln) variant, identifying 388 autosomal differentially methylated regions (DMRs) (for an example DMR, see Supplemental Fig. S5), all characterized by hypomethylation in *DNMT3A* c.2312G > A; p.(Arg771Gln) carriers apart from one 739-bp DMR that showed increased DNA methylation (Supplemental Table S3). The mean size of the identified DMRs was 625 bp (range = 6–5522 bp), spanning an average of six probes (Supplemental Fig. S6).

We next investigated whether DNMT3A p.(Arg771Gln)-associated DMPs are enriched in specific genic locations (see Methods). We found a modest enrichment of DMPs in regions ≥1500 bp upstream of the transcriptional start site (chi-squared Yates-corrected *P*-value = 0.047) and more prominent enrichment in intergenic regions (chi-squared Yates corrected *P*-value = 1.54 × 10^−14^) (Supplemental Fig. S7). DMPs were also significantly enriched in CpG island shore regions (chi-squared Yates-corrected *P*-value = 8 × 10^−30^) (Supplemental Fig. S7). We also examined DMP occurrence in experimentally determined cancer and reprogramming-specific DMR locations (Doi et al. 2009), finding a 2.4-fold and 4.7-fold overrepresentation (chi-squared Yates-corrected *P*-value = 4.24 × 10^−6^ and chi-squared Yates-corrected *P*-value <1.3 × 10^−41^, respectively), as well as predicted enhancer elements that showed a 1.4-fold overrepresentation (chi-squared Yates-corrected *P*-value = 7.57 × 10^−16^). We then undertook Gene Ontology analysis, accounting for the background distribution of probes on the Illumina 450K array, to functionally annotate the DNA methylation differences observed in the *DNMT3A* c.2312G > A; p.(Arg771Gln) carriers. The 2606 DMPs identified in this study showed a significant overrepresentation in functional pathways related to morphogenesis, development, and differentiation (top hit GO:0007275, multicellular organism development, contains 474 genes associated with DMPs; FDR *Q*-value = 3.7 × 10^−7^) (Fig. 1C; Supplemental Table S4). We also performed a functional overlap analysis to identify cell- or tissue-specific chromatin signals associated with these DMPs using eFORGE (Breeze et al. 2016). Significant overlap (FDR *Q*-value < 0.01) was found with DNase I sensitivity hotspots, most apparent with pluripotent cells in ENCODE (The ENCODE Project Consortium 2012; Davis et al. 2018) and fetal tissues within the NIH Roadmap Epigenomics Consortium data set (Supplemental Data S1; Roadmap Epigenomics Consortium et al. 2015). Chromatin states from the NIH Roadmap Epigenomics Consortium data set show an enrichment of DMPs in regions defined as active transcriptional start sites in brain tissue and embryonic stem cells. Of particular interest, given the established importance of DNMT3A during embryonic development, eFORGE analysis of blood cell types highlighted an enrichment of DMPs in regions characterized by repressed Polycomb and enhancer activity.

To provide additional evidence to support the notion that *DNMT3A* c.2312G > A; p.(Arg771Gln) carriers show disruption to developmental pathways, we used the Genomic Regions Enrichment of Annotation Tool (GREAT) (McLean et al. 2010) to explore functional pathways enriched in genes annotated to *DNMT3A* c.2312G > A; p.(Arg771Gln)–associated DMRs. This revealed a significant effect on genes implicated in developmental pathways (first ranked GO biological process = skeletal system development, fold enrichment = 2.5, binomial FDR *Q*-value = 9.22 × 10^−7^), with a specific enrichment for Homeobox protein domain encoding genes (InterPro; fold enrichment = 239.59, binomial FDR *Q*-value = 4.15 × 10^−23^), fundamental for normal developmental processes. An enrichment for malignancy terms was also noted (from the Molecular Signatures Database; first ranked term = Genes with promoters occupied by PML-RARA fusion protein in acute promyelocytic leukemia [APL] cells NB4 and two APL primary blasts, based on ChIP-seq data, fold enrichment = 3.14, binomial FDR *Q*-value = 6.18 × 10^−7^) (see Supplemental Data S2; Liberzon et al. 2011).

**Figure 2.**
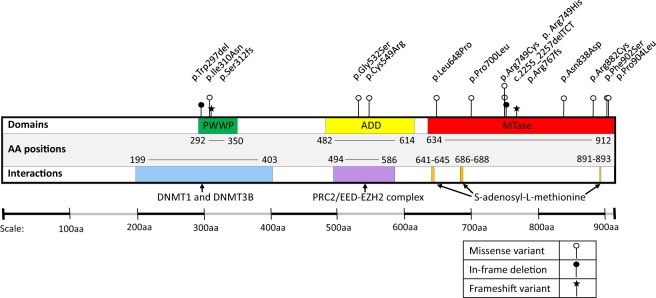
Schematic representation of DNMT3A. The positions of the disease-associated variants included in this study are indicated relative to the protein domain architecture. PWWP, proline-tryptophan-tryptophan-proline domain; ADD, ATRX-Dnmt3-Dnmt3L domain; MTase, Methyltransferase domain; AA, amino acid.

To establish whether these DMPs are a consistent feature of TBRS, we profiled a further 15 non-Amish patients carrying distinct previously published *DNMT3A* pathogenic variants (Table 1; Fig. 2) using the Illumina EPIC DNA methylation array. Examination of the DMPs identified in *DNMT3A* c.2312G > A; p.(Arg771Gln) carriers revealed that the majority of DMPs were common to all of the TBRS patients regardless of the underlying causative *DNMT3A* variant (Fig. 1D), with a Pearson correlation coefficient of 0.6620 (*P*-value <2.2 × 10^−16^) for effect sizes across all DMPs. Each variant showed some heterogeneity in effect size (Fig. 1E), with *DNMT3A* c.2644C > T p.(Arg882Cys) associated with the greatest overall changes in DNA methylation. This data leads us to conclude that TBRS patients show loss of methylation at sites annotated to key genes involved in development and growth pathways, mirroring the well-characterized overgrowth and neurocognitive features that characterize this disorder.

**Table 1.**
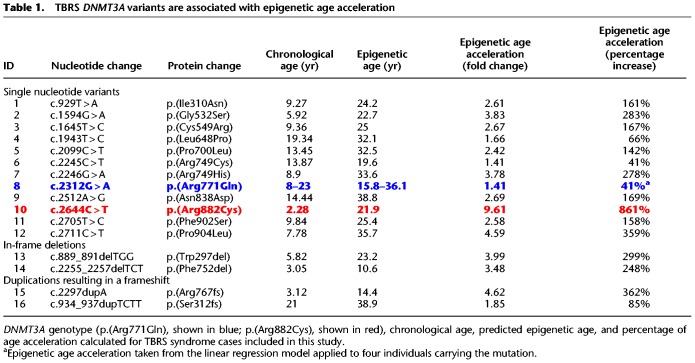
TBRS *DNMT3A* variants are associated with epigenetic age acceleration

### *DNMT3A* mutations are associated with highly accelerated epigenetic aging, particularly the cardinal AML driver mutation p.Arg882Cys

**Figure 3.**
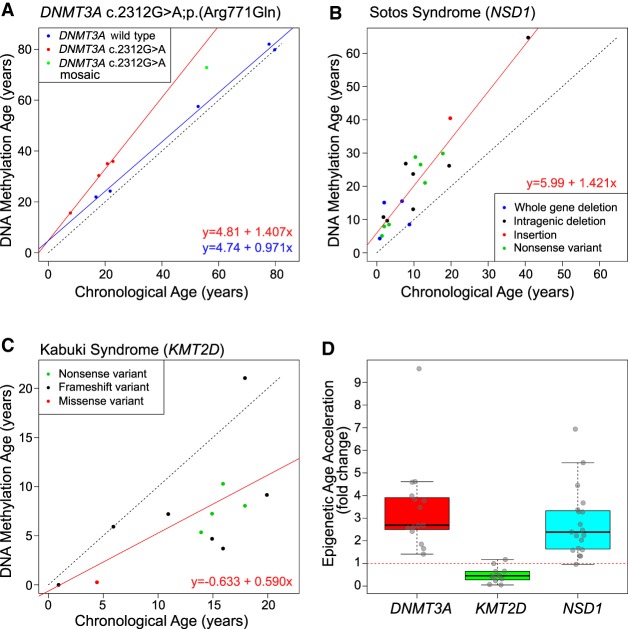
Altered epigenetic aging is observed in methyltransferase-associated human growth disorders. (*A*) Scatter plot comparing “DNA methylation age” derived from the Illumina 450K data (*y*-axis) and actual chronological age (*x*-axis) in *DNMT3A* c.2312G > A p.(Arg771Gln) pathogenic variant carriers (red) versus wild-type family members (blue). Green indicates the mosaic individual. The linear regression model is also shown. (*B*) Scatter plot comparing DNA methylation age versus chronological age in patients with Sotos syndrome. In-frame legend illustrates the different *NSD1* pathogenic variants studied. (*C*) Scatter plot comparing DNA methylation age versus chronological age in patients with Kabuki syndrome. In-frame legend illustrates the different *KMT2D* pathogenic variants studied. (*D*) Boxplot comparing the epigenetic age acceleration rates found in association with TBRS *DNMT3A* variants, *KMT2D* Kabuki syndrome variants, and *NSD1* Sotos syndrome variants. Each age acceleration observation is plotted as a circle. The dotted red line denotes no age acceleration.

DNA methylation at a specific set of CpG sites, representing a so-called “epigenetic clock,” has been shown to be strongly correlated with chronological age (Horvath 2013). Deviations from chronological age have been associated with several measures of accelerated biological aging and age-related phenotypes (Johnson et al. 2012; Levine et al. 2015; Marioni et al. 2015; Chen et al. 2016). We investigated the DNA methylation age of *DNMT3A* c.2312G > A p.(Arg771Gln) carriers using the DNA age calculator (Horvath 2013; http://dnamage.genetics.ucla.edu/), finding that *DNMT3A* c.2312G > A; p.(Arg771Gln) carriers show evidence for highly accelerated aging—an increase of ∼40% beyond their chronological age—compared with wild-type family members (ANCOVA *P*-value = 0.004) (Fig. 3A). Only one of 353 probes used in the epigenetic clock (Horvath 2013) overlapped with the DMPs significantly associated with the *DNMT3A* c.2312G > A; p.(Arg771Gln) pathogenic variant, leading us to conclude that this finding represented a true acceleration of epigenetic age. Furthermore, compared with an extensive number (322) of wild-type control samples profiled in a previous study from our group (Hannon et al. 2016), *DNMT3A* c.2312G > A; p.(Arg771Gln), carriers were consistent outliers for epigenetic age, suggesting their profiles fall outside the normal distribution of variance observed in the general population (Supplemental Fig. S8). Consistent with this, the mosaic Amish father was found to have an intermediate level of epigenetic age acceleration, with a 23% increase over his chronological age. This age acceleration was a cumulative process as indicated by the increased slope of *DNMT3A* c.2312G > A; p.(Arg771Gln) carriers versus wild-type. Epigenetic age could therefore be predicted by the linear regression model as follows: epigenetic age = 4.81 + 1.405 × chronological age. The cumulative increase of epigenetic age relative to chronological age is also notable compared with a recent meta-analysis of longitudinal cohort data that shows the trajectory of epigenetic age in different populations progresses at a slightly slower rate compared with increasing chronological age (Marioni et al. 2019).

We next looked for evidence of elevated epigenetic aging in TBRS patients carrying one of the 15 additional de novo *DNMT3A* pathogenic variants. All TBRS patients showed accelerated epigenetic aging, although the position and type of each variant result in differing degrees of accelerated epigenetic aging (Table 1). The greatest rate of epigenetic age acceleration (>800%) was observed in association with the germline p.(Arg882Cys) substitution, somatic mutation of DNMT3A Arg882 being the most commonly associated with AML.

### Altered epigenetic aging in methyltransferase-associated human growth disorders

To determine whether altered epigenetic aging is a characteristic of other growth disorders associated with disruption of epigenetic regulatory molecules, we extended our study using publicly available Illumina 450K DNA methylation data. We first analyzed the data from individuals with Sotos syndrome, a congenital overgrowth syndrome that results from mutation of the epigenetic modifier *NSD1* (Supplemental Table S5), a lysine histone methyltransferase (Kurotaki et al. 2002; Qiao et al. 2011). Consistent with *DNMT3A* pathogenic variant carriers, these individuals are characterized by an epigenetic age acceleration of ∼40% (linear regression model R^2^ = 0.869, *P*-value = 6.4 × 10^−9^) (Fig. 3B,D). We then examined data from Kabuki syndrome patients carrying pathogenic variants in the *KMT2D* gene (Supplemental Table S6), which also encodes a lysine histone methyltransferase (Ng et al. 2010; Butcher et al. 2017). Kabuki syndrome is a multisystem disorder. Patients typically present with postnatal growth deficiency (rather than overgrowth), a characteristic facial gestalt, ID, and other variable phenotypic features. Although there is more heterogeneity in epigenetic age when compared with the *NSD1* pathogenic variant carriers, there was a significant reduction in epigenetic age of ∼40% seen across these individuals (linear regression model R^2^ = 0.418, *P*-value = 0.023) (Fig. 3C,D).

## Discussion

To date, 78 individuals have been described with the overgrowth condition TBRS. Within this group, a wide variety of germline *DNMT3A* pathogenic variants have been reported, including 33 missense, eight stop-gain, seven frameshift and two splice site variants, two in-frame and five whole-gene deletions (including a set of identical twins) (Tatton-Brown et al. 2014; Okamoto et al. 2016; Tlemsani et al. 2016; Hollink et al. 2017; Kosaki et al. 2017; Lemire et al. 2017; Shen et al. 2017; Spencer et al. 2017; Tatton-Brown et al. 2017, 2018; Xin et al. 2017). Clinically, the predominant features of TBRS are overgrowth, a characteristic facial gestalt, and neurocognitive impairment. These features show phenotypic overlap with conditions associated with germline pathogenic variants in other epigenetic regulatory genes, including Sotos and Weaver syndromes caused by variants in *NSD1* and *EZH2* histone methyltransferases, respectively (Tatton-Brown et al. 2017). These genes encode essential epigenetic regulatory proteins, with a dual somatic/germline role in the pathogenesis of hematological malignancies and overgrowth syndromes with variable degrees of intellectual impairment (Tatton-Brown et al. 2014).

The majority of *DNMT3A* pathogenic variants in TBRS have been found to be de novo, with five individuals inheriting the pathogenic variant from two mosaic parents (Tlemsani et al. 2016; Xin et al. 2017) and two individuals inheriting the pathogenic variant from their affected father (Lemire et al. 2017). Extensive studies of the role of *DNMT3A* in hematopoietic stem cell (HSC) differentiation are also reported, including the regular occurrence of somatic *DNMT3A* variants in patients with acute myeloid leukemia (AML). The most common somatic pathogenic variant reported in patients with AML affects the amino acid residue Arg882. To date, pathogenic variants predicted to affect this residue have been described in the germline of 12 TBRS patients, five with p.(Arg882His) and seven with p.(Arg882Cys) (Tlemsani et al. 2016; Hollink et al. 2017; Kosaki et al. 2017; Shen et al. 2017; Spencer et al. 2017; Tatton-Brown et al. 2018). Despite these studies, the underlying biological mechanism and outcomes of *DNMT3A* gene mutation in TBRS, as well as the potential risks of hematological malignancy, remain largely unclear.

Here we investigated variation in DNA methylation associated with a germline heterozygous *DNMT3A* missense pathogenic variant c.2312G > A; p.(Arg771Gln), affecting the catalytic MTase domain, in a large Amish family comprising four children with TBRS, unaffected siblings, and their mosaic father who displayed an intermediate clinical phenotype (Xin et al. 2017). Affected individuals were characterized by widespread hypomethylation, with DMPs enriched in the vicinity of genes/regulatory regions associated with growth and development, tissue morphogenesis, and differentiation. The magnitude of hypomethylation typically exceeded 10%, a level often considered to show biological significance (Leenen et al. 2016). The accelerated epigenetic age observed did not appear to be driven by overlap of the *DNMT3A* c.2312G > A; p.(Arg771Gln) variant–associated DMPs with the probes that comprise the epigenetic clock as shown by overlap with only one out of the 353 probes used in the epigenetic age estimation (Horvath 2013). Although the relevance of blood cells to understanding the etiology of TBRS is not yet known, we hypothesize that our findings will be generalizable across cell types given the ubiquitous developmental expression of DNMT3A and given that many age-associated DMPs are shared across different cell types (Zhu et al. 2018). Nevertheless, it would still be prudent to undertake epigenetic age assessment of other tissues from TBRS patients to determine whether epigenetic age truly is accelerated across all cell types or a finding that is limited to blood.

Although dysregulation of growth control has been linked to numerous developmental disorders and malignancy, the specific molecular basis of this relationship is not fully understood. The assessment of DMPs associated with the *DNMT3A* c.2312G >A; p.(Arg771Gln) variant identified an enrichment of pluripotent and fetal DNase I sensitivity hotspots, as well as brain and embryonic stem cell–associated chromatin sites according to The ENCODE Project Consortium and Epigenomic Roadmap Consortium data sets (Breeze et al. 2016). Similarly, functional annotation based on Gene Ontology terms showed an overrepresentation of pathways related to morphogenesis, development, and differentiation annotations. DNMT3A loss of function has previously been reported to result in up-regulated multipotency genes and impaired differentiation of neural stem cells and HSCs (Wu et al. 2010; Challen et al. 2011; Jeong et al. 2018) compared with a gain-of-function DNMT3A variant that may increase cellular differentiation (Heyn et al. 2019). It is thus conceivable that TBRS-associated DNMT3A variants may promote increased proliferation of stem/progenitor cell pool, resulting in increased cell numbers during organ morphogenesis and clinical overgrowth.

Our finding of altered epigenetic outcomes in TBRS prompted us to consider similar investigations in other growth disorders associated with epigenetic dysfunction: Sotos syndrome, a neurodevelopmental disorder with features overlapping TBRS and with association with overgrowth in childhood owing to histone methyltransferase *NSD1* gene alterations, and Kabuki syndrome, a distinct neurodevelopmental disorder associated with poor growth and histone methyltransferase *KMT2D* gene alterations. This work defined clear aberrations in epigenetic aging appropriate to the specific nature of each condition. In both overgrowth conditions, TBRS and Sotos syndrome, we identified accelerated epigenetic aging as measured by the DNA methylation age calculator (Horvath 2013). Conversely, patients with Kabuki syndrome, clinically characterized by poor growth, displayed decelerated epigenetic age. Epigenetic age has been strongly correlated with chronological age in unaffected individuals in previous studies of a variety of tissue types (Hannum et al. 2013; Horvath 2013). The observation of accelerated epigenetic aging in both TBRS and Sotos syndrome potentially results from reduced methyltransferase activity in addition to increased cell turnover associated with the overgrowth seen with these disorders, with the converse being the case for Kabuki syndrome. Accelerated epigenetic aging has been associated with age-related clinical characteristics and mortality in epidemiological studies. For example, accelerated epigenetic age in lymphocytes correlates with reduced physical and cognitive function in the elderly and with increased overall mortality independent of other variables such as BMI, sex, and smoking status (Marioni et al. 2015; Chen et al. 2016). The molecular basis of TBRS has only been determined relatively recently, and as such, most of the affected individuals reported are children and young adults. There is therefore still only very limited data available relating to the progression and prognosis of this disorder, meaning that it is not yet possible to determine whether there might be any clinical evidence of multimorbidity indicative of premature aging or a reduction in average life span in TBRS. Further long-term natural history studies of TBRS patients will be extremely helpful for determining the clinical implications of the epigenetic age acceleration observed as a feature of this disorder.

Accelerated epigenetic age has previously been reported in association with specific diseases such as Huntington's disease (+3.4 yr) (Horvath et al. 2016), Down syndrome (+6.6 yr) (Horvath et al. 2015), and Werner's syndrome (+6.4 yr) (Maierhofer et al. 2017). The accelerated epigenetic aging described in association with these disorders is an average increase in epigenetic age, which is relatively consistent throughout lifespan. A distinguishing feature of carriers of the Amish *DNMT3A* c.2312G > A; p.(Arg771Gln) variant is the year-on-year or cumulative increase of accelerated epigenetic aging over life time course, in other words, a true acceleration of epigenetic aging. Although it was not possible to undertake these studies for other *DNMT3A* variants, this may be indicative of a similar effect on cumulative epigenetic age acceleration over the life course in TBRS. It is also noted that the gene encoding DNMT3L is located on Chromosome 21; given the previous report of an average DNA methylation age acceleration of 6.6 yr in blood and brain tissue in individuals with Down syndrome (Horvath et al. 2015) and the role of DNMT3L in stimulating DNMT3A de novo methylation, further investigations are needed to explore the potential relevance of this observation.

There are currently only four reported cases of an AML tumor carrying the DNMT3A p.Arg771Gln substitution. Biochemical measurements of DNMT3A show that mutations at both the Arg771 and Arg882 residues result in reduced methyltransferase activity, with a greater degree of reduction resulting from Arg882 variants compared with Arg771 variants (2.4-fold difference) (Holz-Schietinger et al. 2012). Given this reduced methyltransferase activity, we may expect to observe more pronounced changes in DNA methylation in patients with germline variants affecting Arg882 compared with variants affecting other amino acid residues such as Arg771. Our data reflected this notion, with alteration Arg882 displaying markedly greater methylation changes compared with the other DNMT3A mutations investigated in this study. Currently, available literature suggests that the risk of hematological malignancy in TBRS individuals may vary depending on the specific pathogenic variant underling their condition (Hollink et al. 2017). The significantly advanced epigenetic age that we observed in association with p.Arg882Cys may explain why hematological malignancy has to date only been reported in two TBRS patients, one harboring this germline variant and the second the p.Tyr735Ser variant, the latter not being assessed in this study (Hollink et al. 2017; Tatton-Brown et al. 2018).

In summary, our findings identify widespread DNA hypomethylation in genes involved in morphogenesis, development, differentiation, and malignancy in TBRS patients. TBRS patients also displayed highly accelerated DNA methylation aging. Our studies additionally defined phenotype-related altered epigenetic aging in two histone methyltransferase disorders: *NSD1* Sotos syndrome overgrowth disorder and *KMT2D* Kabuki syndrome growth impairment. Taken together, these findings provide important new insights into the role of DNMT3A during development and of relevance to hematological malignancy, and define perturbation to epigenetic machinery and biological aging as common themes in overgrowth and growth deficiency syndromes.

## Methods

### Genetic and clinical studies

The phenotypic features of the four affected siblings (three females and one male, aged 10–25 yr) (Fig. 1A, individuals III:3, III:5, III:7, and III:13) include macrocephaly, tall stature, hypotonia, mild to moderate ID, behavioral problems, and a distinctive facial appearance. Whole-genome SNP genotyping and exome sequencing of DNA samples taken with informed consent under regionally approved protocols excluded pathogenic variants in known genes, or candidate new genes, associated with neurodevelopmental disorders. Subsequent studies defined a heterozygous c.2312G > A variant in *DNMT3A,* resulting in a p.(Arg771Gln) substitution, as the cause of the condition. Full clinical details are previously described (Xin et al. 2017). Further testing revealed mosaicism for the *DNMT3A* c.2312G > A variant in the father, and Xin et al. (2017) showed pathogenic variant load varied in different tissue types.

### DNA methylation profiling

Genomic DNA from blood was sodium bisulfite converted using the EZ-96 DNA methylation kit (Zymo Research) and DNA methylation quantified across the genome using the Illumina Infinium HumanMethylation450 array (Illumina 450K array) (Illumina). The additional 15 *DNMT3A* pathogenic variants were profiled using the Illumina Infinium EPIC array (Illumina). The Bioconductor package *wateRmelon* (Pidsley et al. 2013) in R 3.4.1 (R Core Team 2017) was used to import IDAT files, and after checking for suitable sodium bisulfite conversion (bisulfite control probe median >90%), the DNA methylation data were imported and quantile normalized using the *dasen* function in *wateRmelon* and methylation beta values produced (ratio of intensities for methylated versus unmethylated alleles). Probes showing a detection *P*-value >0.05 in at least 1% of samples or a beadcount <3 in 5% of samples were removed across all samples. Any samples showing low quality, indicated by a detection *P*-value >0.05 in ≥1% of probes within a sample, were removed from analysis. Probes containing common SNPs within 10 bp of the CpG site were removed (minor allele frequency >5%). Nonspecific probes and probes on the sex chromosomes were also removed (Chen et al. 2013; Price et al. 2013).

### Identification of DMPs

DMPs were identified using a *limma*-based linear model based on pathogenic variant genotype and sex as a covariate (Smyth 2004) and a Benjamini–Hochberg FDR of 5% applied (Benjamini and Hochberg 1995). When the epigenetic age was used as a covariate, a similar level of DMPs were detected (2557 DMPs) with an 80% overlap to the *limma* model without age as a covariate. Changes in methylation were calculated based on comparison between *DNMT3A* c.2312G > A; p.(Arg771Gln) carriers versus wild-type individuals in the Amish pedigree. The additional 15 *DNMT3A* pathogenic variants were assessed relative to seven wild-type control samples run on the same EPIC array run. Blood cell counts were unknown and so were estimated using the DNA methylation age calculator (Horvath 2013; Koestler et al. 2013) and assessed in the linear model. To identify DMPs, the package DMRcate was used with the same *limma*-based design (Peters et al. 2015).

### Gene Ontology and functional enrichment analyses

Gene Ontology enrichment analysis was performed using genes annotated to FDR corrected DMPs using the *gometh* function of the *missMethyl* package (Phipson et al. 2016), which takes into account potential bias of probe distributions on the beadchip array. KEGG pathway analysis was performed using the *gsameth* command of *missMethyl* and KEGG annotation files from the Bioconductor KEGGREST package (http://bioconductor.org/packages/release/bioc/html/KEGGREST.html). Regional enrichment analysis based on Illumina annotations was performed using a chi-squared test with Yates correction in R. DMRs were functionally annotated using the webtool GREAT (http://great.stanford.edu/public/html/). The top *P*-value–ranked 1000 DMPs were also annotated using the eFORGE tool (https://eforge.altiusinstitute.org/) to perform functional overlap analysis for identifying any cell- or tissue-specific epigenetic signals.

### Quantification of global DNA methylation

Global DNA methylation measurements were made using the luminometric methylation assay (LUMA) (Karimi et al. 2006) based on cleavage by a methylation-sensitive restriction enzyme followed by polymerase extension assay via pyrosequencing on the PyroMark Q24 (Qiagen). Peak heights were obtained using the Pyro Q24 CpG 2.0.6 software and a *t*-test applied in R 3.4.1. Global methylation estimates from the Illumina 450K array were assessed through R 3.4.1 using summary statistics and a Wilcoxon rank-sum test on two pairs of samples matched for age and sex.

### DNA methylation age estimation

Epigenetic age calculations were made using the DNA methylation age calculator (https://dnamage.genetics.ucla.edu/) for Illumina 450K data, and Illumina EPIC arrays were assessed using the *agep* function of the *wateRmelon* Bioconductor package, the latter based on the original calculator developed by Steve Horvath (2013). Accelerated age was calculated for the Amish TBRS *DNMT3A* c.2312G > A; p.(Arg771Gln) carriers and wild-type family members and Sotos syndrome and Kabuki syndrome patients and compared with data from 322 control individuals taken from a previous study (Hannon et al. 2016), using linear models of recorded chronological age and calculated epigenetic age. Estimates of age acceleration for the additional 15 TBRS cases were calculated by dividing the calculated epigenetic age with their chronological age. Additional *NSD1* Sotos syndrome patient Illumina DNA methylation files were obtained from GEO accession GSE74432, with corresponding chronological ages derived from the associated paper (Choufani et al. 2015). *KMT2D* Kabuki syndrome DNA methylation data and chronological age were obtained from GEO accession GSE97362 (Butcher et al. 2017).

### Validation of DMPs using bisulfite-pyrosequencing

Bisulfite pyrosequencing was used to validate specific differentially methylated CpG sites originally identified using the Illumina 450K array. Primers, designed using PyroMark Assay Design software (Qiagen), and PCR conditions are provided in Supplemental Table S7. Bisulfite conversion was performed on ∼500 ng of DNA using a bisulfite-gold kit (Zymo Research). PCR was performed with HOT FIREPol DNA polymerase (Solis Biodyne) for 15 min at 95°C followed by 37 cycles of 15 sec at 95°C, 15 sec at annealing temperature (shown in Supplemental Table S7), and 30 sec at 72°C. A final extension of 10 min at 72°C was then applied. DNA methylation was then assessed using the resulting bisulfite PCR amplicons, together with a pyrosequencing primer on the PyroMark Q24 system (Qiagen) following the manufacturer's standard instructions and the Pyro Q24 CpG 2.0.6 software.

## Data access

The raw and processed primary data sets generated in this study have been submitted to the NCBI Gene Expression Omnibus (GEO; https://www.ncbi.nlm.nih.gov/geo/) under accession number GSE128801. R scripts are provided as Supplemental Code S1 and at the following repository: https://github.com/arjeffries/TBRS2019.

## Supplementary Material

Supplemental Material
