# Supplementary material for: Growth disrupting mutations in epigenetic regulatory molecules are associated with abnormalities of epigenetic aging
Source: Genome Res. 2019 Jul;29(7):1057–66. doi: 10.1101/gr.243584.118 (PMC6633263; doi:10.1101/gr.243584.118)
Supplement: Supplemental Material [file supp_29_7_1057__index.html]

Growth disrupting mutations in epigenetic regulatory molecules are associated with abnormalities of epigenetic aging — Supplemental Material 

# Growth disrupting mutations in epigenetic regulatory molecules are associated with abnormalities of epigenetic aging

## Supplemental Material

- Supplemental\_Table\_S1.xlsx
- Supplemental\_Table\_S3.xlsx
- Supplemental\_Table\_S4.xlsx
- Supplemental\_Data\_S1.pdf
- Supplemental\_Data\_S2.pdf
- Supplemental\_Code\_S1.txt
- Supplemental\_Material.pdf
