# Supplementary material for: Growth disrupting mutations in epigenetic regulatory molecules are associated with abnormalities of epigenetic aging
Source: Genome Res. 2019 Jul;29(7):1057–66. doi: 10.1101/gr.243584.118 (PMC6633263; doi:10.1101/gr.243584.118)
Supplement: Supplemental Material [file supp_gr.243584.118_Supplemental_Data_S1.pdf]

DMPs in DNase I sites (probably TF sites) in cell lines for encode Top_1000_DMPs

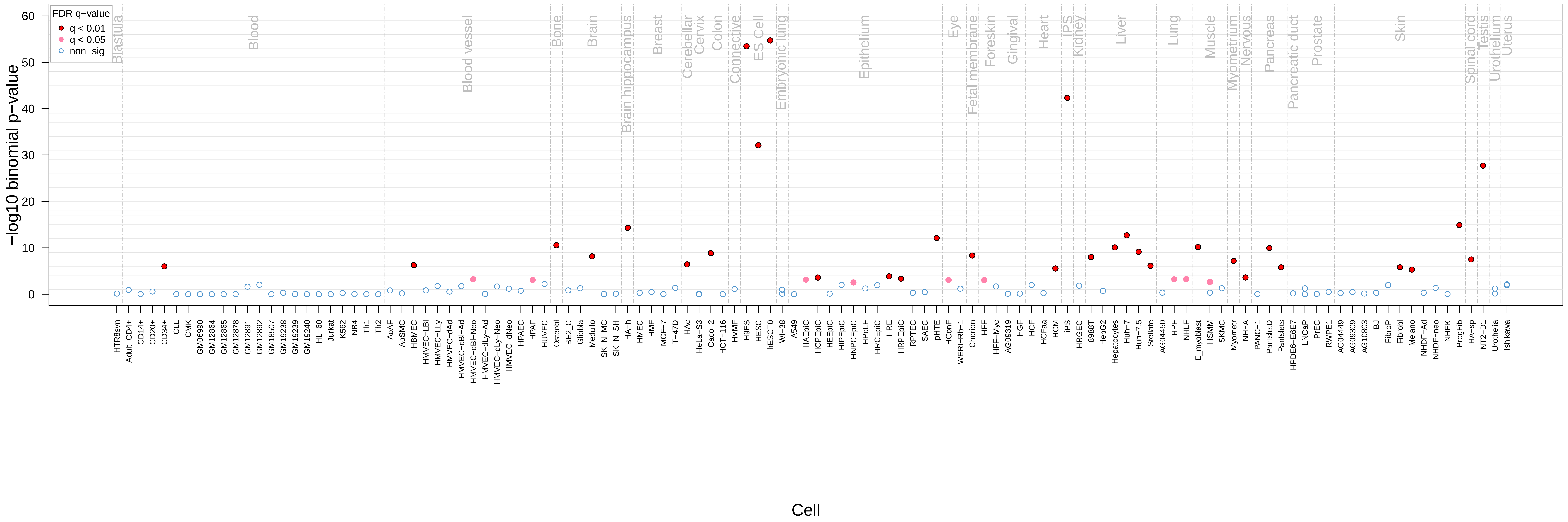

DMPs in DNase I sites (probably TF sites) in cell lines for erc2−DHS Top_1000_DMPs

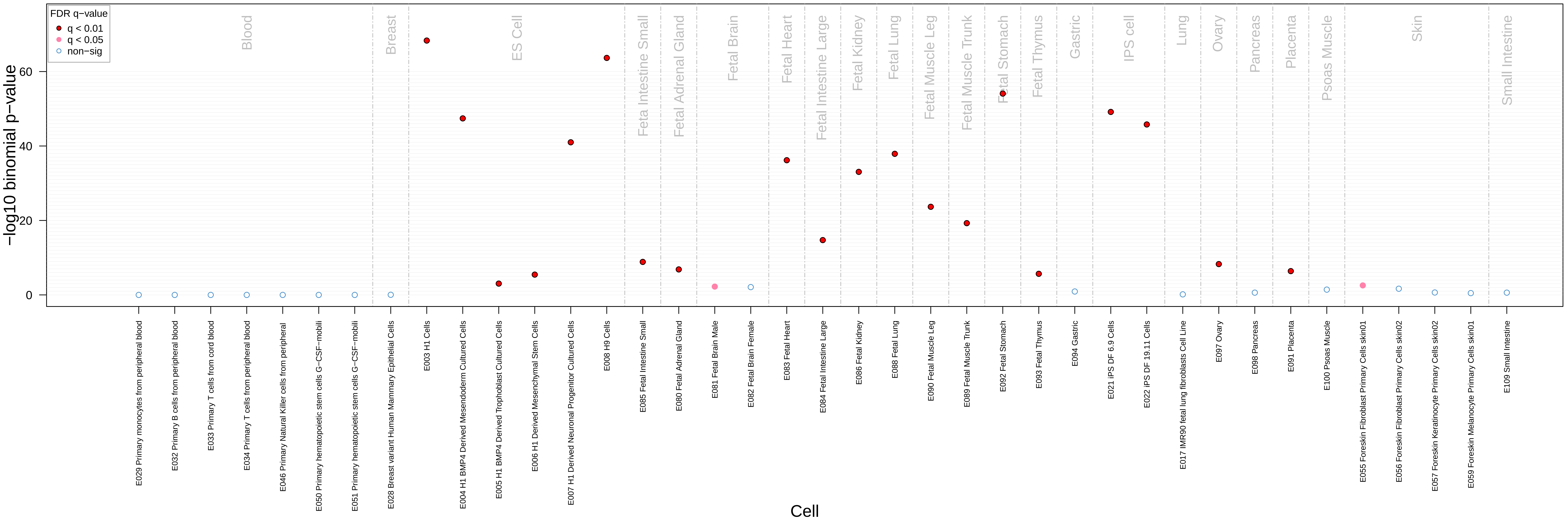

DMPs in DNase I sites (probably TF sites) in cell lines for erc2−chromatin15state−all Top_1000_DMPs

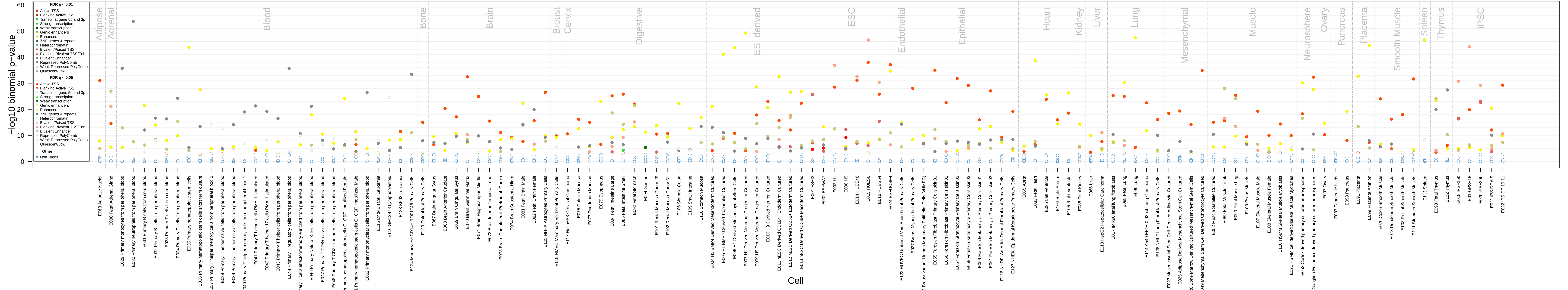

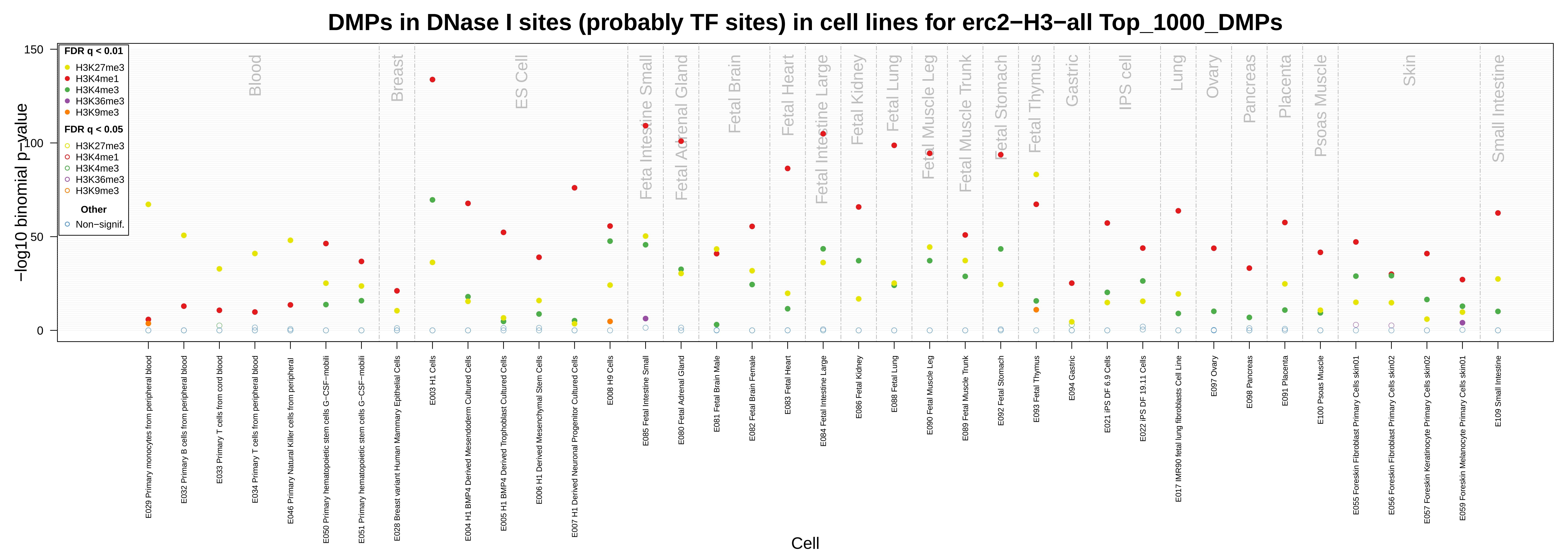
DMPs in DNase I sites (probably TF sites) in cell lines for erc2−H3−all Top_1000_DMPs
−log10 binomial p−value
Cell
FDR q < 0.01
H3K27me3
H3K4me1
H3K4me3
H3K36me3
H3K9me3
FDR q < 0.05
H3K27me3
H3K4me1
H3K4me3
H3K36me3
H3K9me3
Other
Non−signif.
Blood
Breast
ES Cell
Feta Intestine Small
Fetal Adrenal Gland
Fetal Brain
Fetal Heart
Fetal Intestine Large
Fetal Kidney
Fetal Lung
Fetal Muscle Leg
Fetal Muscle Trunk
Fetal Stomach
Fetal Thymus
Gastric
IPS cell
Lung
Ovary
Pancreas
Placenta
Psoas Muscle
Skin
Small Intestine
E029 Primary monocytes from peripheral blood
E032 Primary B cells from peripheral blood
E033 Primary T cells from cord blood
E034 Primary T cells from peripheral blood
E046 Primary Natural Killer cells from peripheral
E050 Primary hematopoietic stem cells G−CSF−mobili
E051 Primary hematopoietic stem cells G−CSF−mobili
E028 Breast variant Human Mammary Epithelial Cells
E003 H1 Cells
E004 H1 BMP4 Derived Mesendoderm Cultured Cells
E005 H1 BMP4 Derived Trophoblast Cultured Cells
E006 H1 Derived Mesenchymal Stem Cells
E007 H1 Derived Neuronal Progenitor Cultured Cells
E008 H9 Cells
E085 Fetal Intestine Small
E080 Fetal Adrenal Gland
E081 Fetal Brain Male
E082 Fetal Brain Female
E083 Fetal Heart
E084 Fetal Intestine Large
E086 Fetal Kidney
E088 Fetal Lung
E090 Fetal Muscle Leg
E089 Fetal Muscle Trunk
E092 Fetal Stomach
E093 Fetal Thymus
E094 Gastric
E021 iPS DF 6.9 Cells
E022 iPS DF 19.11 Cells
E017 IMR90 fetal lung fibroblasts Cell Line
E097 Ovary
E098 Pancreas
E091 Placenta
E100 Psoas Muscle
E055 Foreskin Fibroblast Primary Cells skin01
E056 Foreskin Fibroblast Primary Cells skin02
E057 Foreskin Keratinocyte Primary Cells skin02
E059 Foreskin Melanocyte Primary Cells skin01
E109 Small Intestine
0
50
100
150
