# Supplementary material for: Growth disrupting mutations in epigenetic regulatory molecules are associated with abnormalities of epigenetic aging
Source: Genome Res. 2019 Jul;29(7):1057–66. doi: 10.1101/gr.243584.118 (PMC6633263; doi:10.1101/gr.243584.118)
Supplement: Supplemental Material [file supp_gr.243584.118_Supplemental_Data_S2.pdf]

GREAT version 3.0.0 current (02/15/2015 to now)

Job Description

Region-Gene Association Graphs

What do these graphs illustrate?

Number of associated genes per region

Download as PDF.

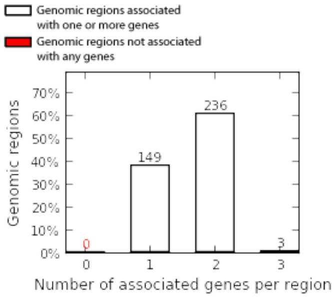

Binned by orientation and distance to TSS

Download as PDF.

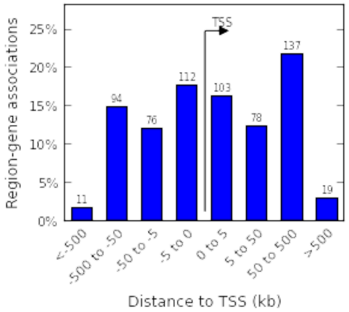

Binned by absolute distance to TSS

Download as PDF.

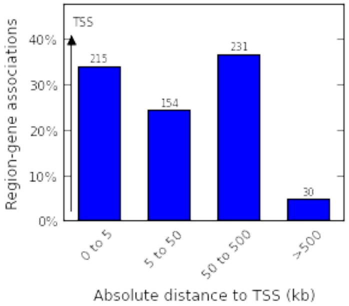

Global Controls

Global Export

Which data is exported by each option?

GO Molecular Function (3 terms)

Global controls

Table controls: Export Shown top rows in this table: 20 Set Term annotation count: Min: 1 Max: Inf Set Visualize this table: [select one]

Term Name	Term ID	Binom Rank	Binom Raw P-Value	Binom Bonferroni P-Value	*Binom FDR Q-Val*	Binom Fold Enrichment	Binom Expected	Binom Observed Region Hits	Binom Genome Fraction	Binom Region Set Coverage	Hyper Rank	Hyper Raw P-Value	Hyper Bonferroni P-Value	*Hyper FDR Q-Val*	Hyper Fold Enrichment	Hyper Expected	Hyper Observed Gene Hits	Hyper Total Genes	Hyper Gene Set Coverage	Hyper Term Gene Coverage
**sequence-specific DNA binding**	GO:0043565	2	**5.7476e-10**	**2.1197e-6**	**1.0598e-6**	2.0959	36.2619	76	0.0935	19.59%	2	**4.7470e-17**	**1.7507e-13**	**8.7535e-14**	3.1263	21.7511	68	697	12.08%	9.76%
**HMG box domain binding**	GO:0071837	5	**8.0649e-7**	**2.9743e-3**	**5.9486e-4**	7.9439	1.2588	10	0.0032	2.58%	9	**1.7248e-5**	**6.3610e-2**	**7.0678e-3**	10.1193	0.5929	6	19	1.07%	31.58%
**regulatory region DNA binding**	GO:0000975	6	**7.9757e-6**	**2.9414e-2**	**4.9024e-3**	2.0768	20.2232	42	0.0521	10.82%	5	**5.4813e-8**	**2.0215e-4**	**4.0430e-5**	2.8814	11.4529	33	367	5.86%	8.99%

The test set of 388 genomic regions picked 563 (3%) of all 18,041 genes.
GO Molecular Function has 3,688 terms covering 15,090 (84%) of all 18,041 genes, and 189,388 term - gene associations.
3,688 ontology terms (100%) were tested using an annotation count range of [1, Inf].

GO Biological Process (17 terms)

Global controls

Table controls: Export Shown top rows in this table: 20 Set Term annotation count: Min: 1 Max: Inf Set Visualize this table: [select one]

Term Name	Term ID	Binom Rank	Binom Raw P-Value	Binom Bonferroni P-Value	*Binom FDR Q-Val*	Binom Fold Enrichment	Binom Expected	Binom Observed Region Hits	Binom Genome Fraction	Binom Region Set Coverage	Hyper Rank	Hyper Raw P-Value	Hyper Bonferroni P-Value	*Hyper FDR Q-Val*	Hyper Fold Enrichment	Hyper Expected	Hyper Observed Gene Hits	Hyper Total Genes	Hyper Gene Set Coverage	Hyper Term Gene Coverage
**skeletal system development**	GO:0001501	1	**8.8312e-11**	**9.2198e-7**	**9.2198e-7**	2.5437	22.8016	58	0.0588	14.95%	2	**2.9408e-16**	**3.0702e-12**	**1.5351e-12**	3.8962	12.5763	49	403	8.70%	12.16%

Name	ID	Rank	P-Value	Bonferroni P-Value	FDR Q-Val	Fold Enrichment	Expected	Region Hits	Genome Fraction	Set Coverage	Rank	Raw P-Value	Bonferroni P-Value	FDR Q-Val	Fold Enrichment	Expected	Gene Hits	Total Genes	Gene Set Coverage	Gene Coverage
**embryonic morphogenesis**	GO:0048598	2	**5.2786e-10**	**5.5108e-6**	**2.7554e-6**	2.2762	28.5569	65	0.0736	16.75%	4	**2.8600e-15**	**2.9858e-11**	**7.4645e-12**	3.4063	15.8530	54	508	9.59%	10.63%
**anterior/posterior pattern specification**	GO:0009952	3	**6.9152e-10**	**7.2195e-6**	**2.4065e-6**	3.3730	10.3765	35	0.0267	9.02%	18	**2.7401e-12**	**2.8607e-8**	**1.5893e-9**	4.7656	6.0853	29	195	5.15%	14.87%
**embryonic organ development**	GO:0048568	5	**1.8247e-9**	**1.9050e-5**	**3.8101e-6**	2.4148	22.7760	55	0.0587	14.18%	20	**3.1036e-12**	**3.2401e-8**	**1.6201e-9**	3.4333	12.2330	42	392	7.46%	10.71%
**embryonic organ morphogenesis**	GO:0048562	6	**5.4845e-9**	**5.7258e-5**	**9.5431e-6**	2.7034	15.9061	43	0.0410	11.08%	25	**1.4103e-11**	**1.4723e-7**	**5.8893e-9**	3.9754	8.3010	33	266	5.86%	12.41%
**regionalization**	GO:0003002	9	**4.0631e-8**	**4.2419e-4**	**4.7132e-5**	2.5182	17.0755	43	0.0440	11.08%	30	**1.9585e-11**	**2.0447e-7**	**6.8157e-9**	3.7385	9.3620	35	300	6.22%	11.67%
**pattern specification process**	GO:0007389	13	**1.6930e-7**	**1.7675e-3**	**1.3596e-4**	2.1457	24.7002	53	0.0637	13.66%	12	**7.0261e-13**	**7.3353e-9**	**6.1127e-10**	3.4009	13.2316	45	424	7.99%	10.61%
**skeletal system morphogenesis**	GO:0048705	28	**9.7855e-7**	**1.0216e-2**	**3.6486e-4**	2.7370	10.9607	30	0.0282	7.73%	23	**9.3387e-12**	**9.7496e-8**	**4.2390e-9**	4.6976	5.9605	28	191	4.97%	14.66%
**embryonic skeletal system morphogenesis**	GO:0048704	33	**2.2968e-6**	**2.3979e-2**	**7.2664e-4**	3.6094	5.2640	19	0.0136	4.90%	47	**1.9138e-10**	**1.9980e-6**	**4.2511e-8**	6.5545	2.7462	18	88	3.20%	20.45%
**embryonic skeletal system development**	GO:0048706	41	**4.5261e-6**	**4.7252e-2**	**1.1525e-3**	2.9918	7.6876	23	0.0198	5.93%	15	**1.3688e-12**	**1.4290e-8**	**9.5269e-10**	6.2993	3.6512	23	117	4.09%	19.66%
**ossification**	GO:0001503	52	**1.1910e-5**	**1.2434e-1**	**2.3912e-3**	2.6096	9.9634	26	0.0257	6.70%	92	**3.7858e-6**	**3.9524e-2**	**4.2961e-4**	3.2532	6.1477	20	197	3.55%	10.15%
**muscle organ development**	GO:0007517	67	**5.3845e-5**	**5.6215e-1**	**8.3903e-3**	2.2155	13.5410	30	0.0349	7.73%	111	**2.9925e-5**	**3.1241e-1**	**2.8145e-3**	2.6704	8.2386	22	264	3.91%	8.33%
**chondroblast differentiation**	GO:0060591	69	**6.1310e-5**	**6.4007e-1**	**9.2764e-3**	41.0115	0.0732	3	0.0002	0.77%	131	**1.1812e-4**	**1.0000**	**9.4136e-3**	24.0333	0.1248	3	4	0.53%	75.00%
**negative regulation of myeloid cell differentiation**	GO:0045638	83	**1.5678e-4**	**1.0000**	**1.9720e-2**	3.6344	3.3018	12	0.0085	3.09%	143	**1.5114e-4**	**1.0000**	**1.1034e-2**	4.1083	2.4341	10	78	1.78%	12.82%
**positive regulation of steroid biosynthetic process**	GO:0010893	89	**2.3134e-4**	**1.0000**	**2.7137e-2**	9.3321	0.5358	5	0.0014	1.29%	158	**3.8036e-4**	**1.0000**	**2.5133e-2**	10.6815	0.3745	4	12	0.71%	33.33%
**osteoblast development**	GO:0002076	94	**3.2381e-4**	**1.0000**	**3.5963e-2**	5.5549	1.2601	7	0.0032	1.80%	148	**2.3507e-4**	**1.0000**	**1.6582e-2**	8.4327	0.5929	5	19	0.89%	26.32%
**artery morphogenesis**	GO:0048844	103	**4.8435e-4**	**1.0000**	**4.9093e-2**	3.4182	3.2181	11	0.0083	2.84%	172	**6.7334e-4**	**1.0000**	**4.0870e-2**	4.6731	1.4979	7	48	1.24%	14.58%

The test set of 388 genomic regions picked 563 (3%) of all 18,041 genes.
GO Biological Process has 10,440 terms covering 15,441 (86%) of all 18,041 genes, and 950,065 term - gene associations.
10,440 ontology terms (100%) were tested using an annotation count range of [1, Inf].

GO Cellular Component (1 term)

Global controls

Table controls: Export ▾ Shown top rows in this table: 20 Set Term annotation count: Min: 1 Max: Inf Set Visualize this table: [select one] ▾

Term Name	Term ID	Binom Rank	Binom Raw P-Value	Binom Bonferroni P-Value	*Binom FDR Q-Val*	Binom Fold Enrichment	Binom Expected	Binom Observed Region Hits	Binom Genome Fraction	Binom Region Set Coverage	Hyper Rank	Hyper Raw P-Value	Hyper Bonferroni P-Value	*Hyper FDR Q-Val*	Hyper Fold Enrichment	Hyper Expected	Hyper Observed Gene Hits	Hyper Total Genes	Hyper Gene Set Coverage	Hyper Term Gene Coverage
**PcG protein complex**	GO:0031519	4	**2.6739e-5**	**3.3825e-2**	**8.4563e-3**	5.9780	1.5055	9	0.0039	2.32%	2	**2.2406e-5**	**2.8344e-2**	**1.4172e-2**	6.5732	1.2171	8	39	1.42%	20.51%

The test set of 388 genomic regions picked 563 (3%) of all 18,041 genes.
GO Cellular Component has 1,265 terms covering 16,807 (93%) of all 18,041 genes, and 277,316 term - gene associations.
1,265 ontology terms (100%) were tested using an annotation count range of [1, Inf].

Table controls: Export ▾ Shown top rows in this table: 20 Set Term annotation count: Min: 1 Max: Inf Set Visualize this table: [select one] ▾

Term Name	Term ID	Binom Rank	Binom Raw P-Value	Binom Bonferroni P-Value	*Binom FDR Q-Val*	Binom Fold Enrichment	Binom Expected	Binom Observed Region Hits	Binom Genome Fraction	Binom Region Set Coverage	Hyper Rank	Hyper Raw P-Value	Hyper Bonferroni P-Value	*Hyper FDR Q-Val*	Hyper Fold Enrichment	Hyper Expected	Hyper Observed Gene Hits	Hyper Total Genes	Hyper Gene Set Coverage	Hyper Term Gene Coverage
**thoracic vertebral transformation**	MP:0004618	1	**4.4458e-14**	**3.5202e-10**	**3.5202e-10**	11.9222	1.5098	18	0.0039	4.64%	17	**6.0629e-11**	**4.8006e-7**	**2.8239e-8**	8.9012	1.6852	15	54	2.66%	27.78%
**abnormal sternebra morphology**	MP:0004322	2	**2.7539e-11**	**2.1806e-7**	**1.0903e-7**	6.5333	3.2143	21	0.0083	5.41%	22	**2.4197e-10**	**1.9159e-6**	**8.7088e-8**	8.1469	1.8412	15	59	2.66%	25.42%
**abnormal rib development**	MP:0002823	3	**2.8995e-11**	**2.2958e-7**	**7.6527e-8**	11.9491	1.1716	14	0.0030	3.61%	126	**4.7384e-5**	**3.7518e-1**	**2.9776e-3**	7.0097	0.9986	7	32	1.24%	21.88%
**abnormal cervical vertebrae morphology**	MP:0003048	4	**4.5629e-10**	**3.6129e-6**	**9.0323e-7**	4.4541	5.8374	26	0.0150	6.70%	12	**1.0731e-11**	**8.4969e-8**	**7.0808e-9**	6.0254	3.6512	22	117	3.91%	18.80%
**abnormal vertebrae morphology**	MP:0000137	5	**5.8125e-10**	**4.6024e-6**	**9.2047e-7**	2.6774	18.3013	49	0.0472	12.63%	9	**3.5959e-12**	**2.8472e-8**	**3.1636e-9**	3.5506	11.2656	40	361	7.10%	11.08%
**vertebral transformation**	MP:0003036	6	**7.7012e-10**	**6.0978e-6**	**1.0163e-6**	5.4186	3.8756	21	0.0100	5.41%	35	**3.8747e-9**	**3.0680e-5**	**8.7657e-7**	5.4933	3.2767	18	105	3.20%	17.14%
**abnormal rib morphology**	MP:0000150	7	**8.4592e-10**	**6.6980e-6**	**9.5686e-7**	3.0860	12.6378	39	0.0326	10.05%	30	**1.1165e-9**	**8.8401e-6**	**2.9467e-7**	3.7321	7.7705	29	249	5.15%	11.65%
**abnormal thoracic cage morphology**	MP:0004624	8	**9.3369e-10**	**7.3929e-6**	**9.2412e-7**	2.6059	19.1869	50	0.0495	12.89%	8	**2.5109e-12**	**1.9881e-8**	**2.4851e-9**	3.6649	10.6415	39	341	6.93%	11.44%
**abnormal sternum morphology**	MP:0000157	9	**1.1460e-9**	**9.0737e-6**	**1.0082e-6**	3.0522	12.7775	39	0.0329	10.05%	13	**1.1043e-11**	**8.7435e-8**	**6.7258e-9**	4.5111	6.4286	29	206	5.15%	14.08%
**abnormal presacral vertebrae morphology**	MP:0000459	10	**1.4313e-9**	**1.1333e-5**	**1.1333e-6**	3.2783	10.6764	35	0.0275	9.02%	14	**1.2473e-11**	**9.8759e-8**	**7.0542e-9**	4.4893	6.4598	29	207	5.15%	14.01%
**abnormal rib attachment**	MP:0004625	11	**3.4050e-9**	**2.6961e-5**	**2.4510e-6**	4.7393	4.6421	22	0.0120	5.67%	28	**7.1791e-10**	**5.6844e-6**	**2.0302e-7**	6.0716	2.9646	18	95	3.20%	18.95%
**abnormal pectoral girdle bone morphology**	MP:0004508	12	**3.7139e-9**	**2.9407e-5**	**2.4506e-6**	2.7822	15.0958	42	0.0389	10.82%	7	**1.8438e-12**	**1.4599e-8**	**2.0855e-9**	4.4009	7.2712	32	233	5.68%	13.73%
**cervical vertebral transformation**	MP:0004615	14	**6.3805e-9**	**5.0521e-5**	**3.6086e-6**	8.6979	1.4946	13	0.0039	3.35%	40	**8.5275e-9**	**6.7521e-5**	**1.6880e-6**	8.5452	1.4043	12	45	2.13%	26.67%
**abnormal appendicular skeleton morphology**	MP:0009250	15	**7.4373e-9**	**5.8888e-5**	**3.9259e-6**	2.0960	31.9653	67	0.0824	17.27%	3	**6.6323e-14**	**5.2514e-10**	**1.7505e-10**	3.0780	18.1935	56	583	9.95%	9.61%
**increased rib number**	MP:0000480	16	**7.8694e-9**	**6.2310e-5**	**3.8944e-6**	6.9950	2.1444	15	0.0055	3.87%	47	**9.6083e-8**	**7.6079e-4**	**1.6187e-5**	7.8331	1.4043	11	45	1.95%	24.44%
**abnormal kidney venous blood vessel morphology**	MP:0011311	17	**9.0416e-9**	**7.1591e-5**	**4.2113e-6**	42.7745	0.1403	6	0.0004	1.55%	156	**1.1812e-4**	**9.3528e-1**	**5.9954e-3**	24.0333	0.1248	3	4	0.53%	75.00%
**small xiphoid process**	MP:0004680	18	**1.2869e-8**	**1.0189e-4**	**5.6607e-6**	40.2817	0.1490	6	0.0004	1.55%	134	**5.9436e-5**	**4.7061e-1**	**3.5120e-3**	16.0222	0.2497	4	8	0.71%	50.00%
**abnormal vertebral column morphology**	MP:0004703	19	**5.3617e-8**	**4.2454e-4**	**2.2344e-5**	2.1109	27.9499	59	0.0720	15.21%	20	**8.6317e-11**	**6.8346e-7**	**3.4173e-8**	2.7939	17.5382	49	562	8.70%	8.72%
**abnormal cervical atlas morphology**	MP:0004607	20	**5.6137e-8**	**4.4450e-4**	**2.2225e-5**	6.5404	2.1405	14	0.0055	3.61%	71	**1.8171e-6**	**1.4388e-2**	**2.0264e-4**	6.6759	1.4979	10	48	1.78%	20.83%
**abnormal vertebral arch morphology**	MP:0004599	21	**6.7945e-8**	**5.3799e-4**	**2.5618e-5**	4.1628	5.0446	21	0.0130	5.41%	72	**2.0730e-6**	**1.6414e-2**	**2.2797e-4**	4.5778	3.0583	14	98	2.49%	14.29%

The test set of 388 genomic regions picked 563 (3%) of all 18,041 genes.
Mouse Phenotype has 7,918 terms covering 7,524 (42%) of all 18,041 genes, and 541,054 term - gene associations.
7,918 ontology terms (100%) were tested using an annotation count range of [1, Inf].

Human Phenotype (no terms)

Global controls

Term Name	Term ID	Binom Rank	Binom Raw P-Value	Binom Bonferroni P-Value	*Binom FDR Q-Val*	Binom Fold Enrichment	Binom Expected	Binom Observed Region Hits	Binom Genome Fraction	Binom Region Set Coverage	Hyper Rank	Hyper Raw P-Value	Hyper Bonferroni P-Value	*Hyper FDR Q-Val*	Hyper Fold Enrichment	Hyper Expected	Hyper Observed Gene Hits	Hyper Total Genes	Hyper Gene Set Coverage	Hyper Term Gene Coverage

No results meet your chosen criteria.

The test set of 388 genomic regions picked 563 (3%) of all 18,041 genes.
Human Phenotype has 6,146 terms covering 2,822 (16%) of all 18,041 genes, and 244,804 term - gene associations.
6,146 ontology terms (100%) were tested using an annotation count range of [1, Inf].

Disease Ontology (no terms)

Global controls

Table controls: Export ▾ Shown top rows in this table: 20 Set Term annotation count: Min: 1 Max: Inf Set Visualize this table: [select one] ▾

Term Name	Term ID	Binom Rank	Binom Raw P-Value	Binom Bonferroni P-Value	*Binom FDR Q-Val*	Binom Fold Enrichment	Binom Expected	Binom Observed Region Hits	Binom Genome Fraction	Binom Region Set Coverage	Hyper Rank	Hyper Raw P-Value	Hyper Bonferroni P-Value	*Hyper FDR Q-Val*	Hyper Fold Enrichment	Hyper Expected	Hyper Observed Gene Hits	Hyper Total Genes	Hyper Gene Set Coverage	Hyper Term Gene Coverage

No results meet your chosen criteria.

The test set of 388 genomic regions picked 563 (3%) of all 18,041 genes.
Disease Ontology has 2,235 terms covering 7,886 (44%) of all 18,041 genes, and 232,324 term - gene associations.
2,235 ontology terms (100%) were tested using an annotation count range of [1, Inf].

MSigDB Cancer Neighborhood (no terms)

Global controls

Table controls: Export ▾ Shown top rows in this table: 20 Set Term annotation count: Min: 1 Max: Inf Set Visualize this table: [select one] ▾

Term Name	Term ID	Binom Rank	Binom Raw P-Value	Binom Bonferroni P-Value	*Binom FDR Q-Val*	Binom Fold Enrichment	Binom Expected	Binom Observed Region Hits	Binom Genome Fraction	Binom Region Set Coverage	Hyper Rank	Hyper Raw P-Value	Hyper Bonferroni P-Value	*Hyper FDR Q-Val*	Hyper Fold Enrichment	Hyper Expected	Hyper Observed Gene Hits	Hyper Total Genes	Hyper Gene Set Coverage	Hyper Term Gene Coverage

No results meet your chosen criteria.

The test set of 388 genomic regions picked 563 (3%) of all 18,041 genes.
MSigDB Cancer Neighborhood has 427 terms covering 4,758 (26%) of all 18,041 genes, and 42,051 term - gene associations.
427 ontology terms (100%) were tested using an annotation count range of [1, Inf].

Placenta Disorders (no terms)

Global controls

Table controls: Export ▾ Shown top rows in this table: 20 Set Term annotation count: Min: 1 Max: Inf Set Visualize this table: [select one] ▾

Term Name	Term ID	Binom Rank	Binom Raw P-Value	Binom Bonferroni P-Value	*Binom FDR Q-Val*	Binom Fold Enrichment	Binom Expected	Binom Observed Region Hits	Binom Genome Fraction	Binom Region Set Coverage	Hyper Rank	Hyper Raw P-Value	Hyper Bonferroni P-Value	*Hyper FDR Q-Val*	Hyper Fold Enrichment	Hyper Expected	Hyper Observed Gene Hits	Hyper Total Genes	Hyper Gene Set Coverage	Hyper Term Gene Coverage

No results meet your chosen criteria.

The test set of 388 genomic regions picked 563 (3%) of all 18,041 genes.
Placenta Disorders has 3 terms covering 691 (4%) of all 18,041 genes, and 831 term - gene associations.
3 ontology terms (100%) were tested using an annotation count range of [1, Inf].

PANTHER Pathway (no terms)

Global controls

Term Name	Term ID	Binom Rank	Binom Raw P-Value	Binom Bonferroni P-Value	*Binom FDR Q-Val*	Binom Fold Enrichment	Binom Expected	Binom Observed Region Hits	Binom Genome Fraction	Binom Region Set Coverage	Hyper Rank	Hyper Raw P-Value	Hyper Bonferroni P-Value	*Hyper FDR Q-Val*	Hyper Fold Enrichment	Hyper Expected	Hyper Observed Gene Hits	Hyper Total Genes	Hyper Gene Set Coverage	Hyper Term Gene Coverage

No results meet your chosen criteria.

The test set of 388 genomic regions picked 563 (3%) of all 18,041 genes.
PANTHER Pathway has 152 terms covering 2,197 (12%) of all 18,041 genes, and 5,065 term - gene associations.
152 ontology terms (100%) were tested using an annotation count range of [1, Inf].

BioCyc Pathway (no terms)

Global controls

Table controls: Export ▾ Shown top rows in this table: 20 Set Term annotation count: Min: 1 Max: Inf Set Visualize this table: NEW [select one] ▾

Term Name	Term ID	Binom Rank	Binom Raw P-Value	Binom Bonferroni P-Value	*Binom FDR Q-Val*	Binom Fold Enrichment	Binom Expected	Binom Observed Region Hits	Binom Genome Fraction	Binom Region Set Coverage	Hyper Rank	Hyper Raw P-Value	Hyper Bonferroni P-Value	*Hyper FDR Q-Val*	Hyper Fold Enrichment	Hyper Expected	Hyper Observed Gene Hits	Hyper Total Genes	Hyper Gene Set Coverage	Hyper Term Gene Coverage

No results meet your chosen criteria.

The test set of 388 genomic regions picked 563 (3%) of all 18,041 genes.
BioCyc Pathway has 332 terms covering 1,034 (6%) of all 18,041 genes, and 2,524 term - gene associations.
332 ontology terms (100%) were tested using an annotation count range of [1, Inf].

MSigDB Pathway (no terms)

Global controls

Table controls: Export ▾ Shown top rows in this table: 20 Set Term annotation count: Min: 1 Max: Inf Set Visualize this table: NEW [select one] ▾

Term Name	Term ID	Binom Rank	Binom Raw P-Value	Binom Bonferroni P-Value	*Binom FDR Q-Val*	Binom Fold Enrichment	Binom Expected	Binom Observed Region Hits	Binom Genome Fraction	Binom Region Set Coverage	Hyper Rank	Hyper Raw P-Value	Hyper Bonferroni P-Value	*Hyper FDR Q-Val*	Hyper Fold Enrichment	Hyper Expected	Hyper Observed Gene Hits	Hyper Total Genes	Hyper Gene Set Coverage	Hyper Term Gene Coverage

No results meet your chosen criteria.

The test set of 388 genomic regions picked 563 (3%) of all 18,041 genes.
MSigDB Pathway has 1,320 terms covering 8,117 (45%) of all 18,041 genes, and 62,449 term - gene associations.
1,320 ontology terms (100%) were tested using an annotation count range of [1, Inf].

Term Name	Term ID	Binom Rank	Binom Raw P-Value	Binom Bonferroni P-Value	*Binom FDR Q-Val*	Binom Fold Enrichment	Binom Expected	Binom Observed Region Hits	Binom Genome Fraction	Binom Region Set Coverage	Hyper Rank	Hyper Raw P-Value	Hyper Bonferroni P-Value	*Hyper FDR Q-Val*	Hyper Fold Enrichment	Hyper Expected	Hyper Observed Gene Hits	Hyper Total Genes	Hyper Gene Set Coverage	Hyper Term Gene Coverage

MGI Expression: Detected (20+ terms)

Global controls

Table controls: Export ▾ Shown top rows in this table: 20 Set Term annotation count: Min: 1 Max: Inf Set Visualize this table: NEW [select one] ▾

Term Name	Term ID	Binom Rank	Binom Raw P-Value	Binom Bonferroni P-Value	*Binom FDR Q-Val*	Binom Fold Enrichment	Binom Expected	Binom Observed Region Hits	Binom Genome Fraction	Binom Region Set Coverage	Hyper Rank	Hyper Raw P-Value	Hyper Bonferroni P-Value	*Hyper FDR Q-Val*	Hyper Fold Enrichment	Hyper Expected	Hyper Observed Gene Hits	Hyper Total Genes	Hyper Gene Set Coverage	Hyper Term Gene Coverage
**TS19_T1 vertebral cartilage condensation**	13390	1	**6.3449e-17**	**5.9243e-13**	**5.9243e-13**	422.5308	0.0166	7	4.2698e-5	1.80%	127	**2.3884e-8**	**2.2300e-4**	**1.7559e-6**	24.0333	0.2497	6	8	1.07%	75.00%
**TS19_T2 vertebral cartilage condensation**	13395	2	**1.2054e-16**	**1.1255e-12**	**5.6273e-13**	385.4422	0.0182	7	4.6807e-5	1.80%	148	**6.9756e-8**	**6.5131e-4**	**4.4008e-6**	21.3629	0.2809	6	9	1.07%	66.67%
**TS19_T3 vertebral cartilage condensation**	13400	2	**1.2054e-16**	**1.1255e-12**	**5.6273e-13**	385.4422	0.0182	7	4.6807e-5	1.80%	148	**6.9756e-8**	**6.5131e-4**	**4.4008e-6**	21.3629	0.2809	6	9	1.07%	66.67%

Name	ID	Rank	P-Value	P-Value	Val	Enrichment	Expected	Region Hits	Fraction	Set Coverage	Rank	P-Value	P-Value	Val	Enrichment	Expected	Gene Hits	Genes	Coverage	Gene Coverage
**TS19_T11 vertebral cartilage condensation**	13450	4	**1.8918e-16**	**1.7664e-12**	**4.4160e-13**	361.3513	0.0194	7	4.9927e-5	1.80%	92	**3.0729e-9**	**2.8691e-5**	**3.1186e-7**	22.4311	0.3121	7	10	1.24%	70.00%
**TS19_T5 vertebral cartilage condensation**	13410	4	**1.8918e-16**	**1.7664e-12**	**4.4160e-13**	361.3513	0.0194	7	4.9927e-5	1.80%	92	**3.0729e-9**	**2.8691e-5**	**3.1186e-7**	22.4311	0.3121	7	10	1.24%	70.00%
**TS19_T4 vertebral cartilage condensation**	13405	4	**1.8918e-16**	**1.7664e-12**	**4.4160e-13**	361.3513	0.0194	7	4.9927e-5	1.80%	92	**3.0729e-9**	**2.8691e-5**	**3.1186e-7**	22.4311	0.3121	7	10	1.24%	70.00%
**TS19_T6 vertebral cartilage condensation**	13430	4	**1.8918e-16**	**1.7664e-12**	**4.4160e-13**	361.3513	0.0194	7	4.9927e-5	1.80%	92	**3.0729e-9**	**2.8691e-5**	**3.1186e-7**	22.4311	0.3121	7	10	1.24%	70.00%
**TS19_T7 vertebral cartilage condensation**	13434	4	**1.8918e-16**	**1.7664e-12**	**4.4160e-13**	361.3513	0.0194	7	4.9927e-5	1.80%	92	**3.0729e-9**	**2.8691e-5**	**3.1186e-7**	22.4311	0.3121	7	10	1.24%	70.00%
**TS19_T8 vertebral cartilage condensation**	13438	4	**1.8918e-16**	**1.7664e-12**	**4.4160e-13**	361.3513	0.0194	7	4.9927e-5	1.80%	92	**3.0729e-9**	**2.8691e-5**	**3.1186e-7**	22.4311	0.3121	7	10	1.24%	70.00%
**TS19_T9 vertebral cartilage condensation**	13442	4	**1.8918e-16**	**1.7664e-12**	**4.4160e-13**	361.3513	0.0194	7	4.9927e-5	1.80%	92	**3.0729e-9**	**2.8691e-5**	**3.1186e-7**	22.4311	0.3121	7	10	1.24%	70.00%
**TS19_T10 vertebral cartilage condensation**	13446	4	**1.8918e-16**	**1.7664e-12**	**4.4160e-13**	361.3513	0.0194	7	4.9927e-5	1.80%	92	**3.0729e-9**	**2.8691e-5**	**3.1186e-7**	22.4311	0.3121	7	10	1.24%	70.00%
**TS19_C6 vertebral cartilage condensation**	13368	12	**2.7574e-15**	**2.5746e-11**	**2.1455e-12**	530.8977	0.0113	6	2.9128e-5	1.55%	174	**1.7004e-7**	**1.5877e-3**	**9.1245e-6**	26.7037	0.1872	5	6	0.89%	83.33%
**TS19_C7 vertebral cartilage condensation**	13373	13	**1.0607e-14**	**9.9041e-11**	**7.6185e-12**	423.9541	0.0142	6	3.6475e-5	1.55%	211	**5.7981e-7**	**5.4137e-3**	**2.5657e-5**	22.8889	0.2184	5	7	0.89%	71.43%
**TS19_cervical vertebral cartilage condensation**	3984	14	**1.4604e-12**	**1.3636e-8**	**9.7398e-10**	186.0960	0.0322	6	8.3096e-5	1.55%	282	**6.4344e-6**	**6.0078e-2**	**2.1304e-4**	16.0222	0.3121	5	10	0.89%	50.00%
**TS26_integumental system**	7528	15	**3.5505e-10**	**3.3151e-6**	**2.2101e-7**	3.5434	9.5952	34	0.0247	8.76%	141	**5.6883e-8**	**5.3112e-4**	**3.7668e-6**	3.7412	6.1477	23	197	4.09%	11.68%
**TS26_skin**	14166	17	**9.7630e-10**	**9.1157e-6**	**5.3622e-7**	4.2928	6.0566	26	0.0156	6.70%	180	**2.1483e-7**	**2.0059e-3**	**1.1144e-5**	4.2726	4.2129	18	135	3.20%	13.33%
**TS13_trunk mesenchyme**	498	19	**3.0122e-9**	**2.8125e-5**	**1.4802e-6**	3.2528	10.4526	34	0.0269	8.76%	36	**1.8582e-12**	**1.7350e-8**	**4.8195e-10**	5.0125	5.5860	28	179	4.97%	15.64%
**TS13_trunk paraxial mesenchyme**	503	20	**3.2320e-9**	**3.0177e-5**	**1.5089e-6**	4.1961	5.9579	25	0.0154	6.44%	80	**1.4466e-9**	**1.3507e-5**	**1.6884e-7**	5.8263	3.0895	18	99	3.20%	18.18%
**TS15_limb**	1451	21	**1.1464e-8**	**1.0704e-4**	**5.0969e-6**	2.1879	27.4238	60	0.0707	15.46%	43	**9.3064e-12**	**8.6893e-8**	**2.0208e-9**	3.0612	15.3537	47	492	8.35%	9.55%
**TS25_metanephros**	8015	23	**3.7148e-8**	**3.4685e-4**	**1.5081e-5**	3.1266	9.9151	31	0.0256	7.99%	68	**4.6405e-10**	**4.3328e-6**	**6.3718e-8**	4.1200	6.5534	27	210	4.80%	12.86%

The test set of 388 genomic regions picked 563 (3%) of all 18,041 genes.
MGI Expression: Detected has 9,337 terms covering 11,602 (64%) of all 18,041 genes, and 970,237 term - gene associations.
9,337 ontology terms (100%) were tested using an annotation count range of [1, Inf].

MSigDB Perturbation (20+ terms)

Global controls

Table controls: Export ▾ Shown top rows in this table: 20 Set Term annotation count: Min: 1 Max: Inf Set Visualize this table: [select one] ▾

Term Name	Term ID	Binom Rank	Binom Raw P-Value	Binom Bonferroni P-Value	*Binom FDR Q-Val*	Binom Fold Enrichment	Binom Expected	Binom Observed Region Hits	Binom Genome Fraction	Binom Region Set Coverage	Hyper Rank	Hyper Raw P-Value	Hyper Bonferroni P-Value	*Hyper FDR Q-Val*	Hyper Fold Enrichment	Ex
**Genes with promoters occupied by PML-RARA fusion [GeneID=5371,5914] protein in acute promyelocytic leukemia(APL) cells NB4 and two APL primary blasts, based on Chip-seq data.**	MARTENS_BOUND_BY_PML_RARA_FUSION	1	**1.8373e-10**	**6.1808e-7**	**6.1808e-7**	3.1392	13.0608	41	0.0337	10.57%	3	**1.6222e-9**	**5.4571e-6**	**1.8190e-6**	2.9544	1

Name	ID	Rank	P-Value	[illegible] P-Value	[illegible] *Val*	[illegible] Enrichment	Expected	Region Hits	[illegible] Fraction	Set Coverage	Rank	[illegible] P-Value	[illegible] P-Value	[illegible] *Val*	[illegible] Enrichment	Ex[illegible]
**Genes with high-CpG-density promoters (HCP) bearing the H3K27 tri-methylation (H3K27me3) mark in brain.**	MEISSNER_BRAIN_HCP_WITH_H3K27ME3	2	**4.6380e-9**	**1.5602e-5**	**7.8010e-6**	3.4301	9.0376	31	0.0233	7.99%	6	**5.7355e-8**	**1.9294e-4**	**3.2157e-5**	3.3023	[illegible]
**Genes up-regulated in B-CLL (B-cell chronic lymphocytic leukemia) patients with mutated immunoglobulin variable heavy chain (VH) genes.**	FAELT_B_CLL_WITH_VH_REARRANGEMENTS_UP	3	**3.4497e-8**	**1.1605e-4**	**3.8683e-5**	7.5167	1.7295	13	0.0045	3.35%	14	**1.8171e-6**	**6.1126e-3**	**4.3661e-4**	6.6759	[illegible]
**Genes up-regulated in acute myeloid leukemia (AML) with respect to cellular localization of NPM1 [GeneID=4869]: cytoplasmic vs. nucleolar.**	ALCALAY_AML_BY_NPM1_LOCALIZATION_UP	4	**1.6144e-7**	**5.4308e-4**	**1.3577e-4**	3.5283	6.8021	24	0.0175	6.19%	10	**3.3546e-7**	**1.1285e-3**	**1.1285e-4**	4.1496	[illegible]
**Genes within amplicon 7p15 identified in a copy number alterations study of 191 breast tumor samples.**	NIKOLSKY_BREAST_CANCER_7P15_AMPLICON	5	**2.3136e-7**	**7.7830e-4**	**1.5566e-4**	81.2873	0.0492	4	0.0001	1.03%	49	**2.5997e-4**	**8.7454e-1**	**1.7848e-2**	11.6525	[illegible]
**Top genes associated with unfavorable overall survival of mesothelioma patients after surgery.**	LOPEZ_MESOTHELIOMA_SURVIVAL_OVERALL_DN	6	**4.5845e-7**	**1.5422e-3**	**2.5704e-4**	15.6514	0.4472	7	0.0012	1.80%	79	**9.7346e-4**	**1.0000**	**4.1452e-2**	8.5452	[illegible]
**The 'NPM1-mutated signature 2': genes down-regulated in pediatric AML (acute myeloid leukemia) samples with mutated NPM1 [GeneID=4869] compared to the AML cases with the intact gene and without recurring cytogenetic anomalities or M7 phenotype.**	MULLIGHAN_NPM1_MUTATED_SIGNATURE_2_DN	7	**3.1798e-6**	**1.0697e-2**	**1.5281e-3**	5.4549	2.1999	12	0.0057	3.09%	72	**6.4316e-4**	**1.0000**	**3.0050e-2**	3.7454	[illegible]
**Genes down-regulated in normal hematopoietic progenitors by RUNX1-RUNX1T1 [GeneID=861;862] fusion.**	TONKS_TARGETS_OF_RUNX1_RUNX1T1_FUSION_HSC_DN	8	**3.7644e-6**	**1.2663e-2**	**1.5829e-3**	3.2295	6.5026	21	0.0168	5.41%	20	**1.3088e-5**	**4.4029e-2**	**2.2014e-3**	3.2223	[illegible]
**The 'NPM1 signature 3': genes down-regulated in pediatric AML (acute myeloid leukemia) with mutated NPM1 [GeneID=4869] compared to the AML cases with intact NPM1 and MLL [GeneID=4297].**	MULLIGHAN_NPM1_SIGNATURE_3_DN	9	**6.1446e-6**	**2.0670e-2**	**2.2967e-3**	3.2386	6.1755	20	0.0159	5.15%	22	**1.4035e-5**	**4.7212e-2**	**2.1460e-3**	3.3421	[illegible]
**Genes down-regulated in H358 cells (lung cancer) by inducible expression of CEBPA [GeneID=1050] off plasmid vector.**	HALMOS_CEBPA_TARGETS_DN	10	**7.8105e-6**	**2.6274e-2**	**2.6274e-3**	4.9842	2.4076	12	0.0062	3.09%	19	**1.2445e-5**	**4.1867e-2**	**2.2035e-3**	6.1362	[illegible]
**Genes that best predicted acute myeloid leukemia (AML) with internal tandem duplications (IDT) in FLT3 [GeneID=2322].**	VALK_AML_WITH_FLT3_ITD	12	**1.0102e-5**	**3.3982e-2**	**2.8319e-3**	5.9444	1.6823	10	0.0043	2.58%	41	**1.5107e-4**	**5.0820e-1**	**1.2395e-2**	5.9029	[illegible]
**The 'NPM1-mutated signature 1': genes down-regulated in pediatric AML (acute myeloid leukemia) samples with mutated NPM1 [GeneID=4869] compared to all AML cases with the intact gene.**	MULLIGHAN_NPM1_MUTATED_SIGNATURE_1_DN	15	**2.0587e-5**	**6.9254e-2**	**4.6169e-3**	3.5028	4.5678	16	0.0118	4.12%	43	**1.7091e-4**	**5.7493e-1**	**1.3370e-2**	3.2801	[illegible]
**The 'NPM1-mutated signature 1': genes up-regulated in pediatric AML (acute myeloid leukemia) samples with mutated NPM1 [GeneID=4869] compared to all AML cases with the intact gene.**	MULLIGHAN_NPM1_MUTATED_SIGNATURE_1_UP	16	**2.4882e-5**	**8.3703e-2**	**5.2314e-3**	2.4457	11.0399	27	0.0285	6.96%	16	**4.8181e-6**	**1.6208e-2**	**1.0130e-3**	2.7624	[illegible]
**Genes up-regulated during pubertal mammary gland development between week 4 and 5.**	MCBRYAN_PUBERTAL_BREAST_4_5WK_UP	18	**3.4718e-5**	**1.1679e-1**	**6.4883e-3**	2.3099	12.5549	29	0.0324	7.47%	9	**2.4832e-7**	**8.3534e-4**	**9.2816e-5**	3.1559	[illegible]
**Genes up-regulated in normal bone marrow plasma cells (BMPC) compared to polyclonal plasmablasts (PPC) that also distinguished multiple myeloma (MM) samples by expression of levels of TACI (TNFRSF13B) [GeneID=23495].**	MOREAUX_B_LYMPHOCYTE_MATURATION_BY_TACI_UP	19	**4.5140e-5**	**1.5185e-1**	**7.9922e-3**	4.5139	2.4369	11	0.0063	2.84%	87	**1.1114e-3**	**1.0000**	**4.2972e-2**	3.4747	[illegible]
**Genes down-regulated in mARMS (molecular ARMS) compared to the mERMS (molecular ERMS) class of rhabdomyosarcoma tumors.**	DAVICIONI_MOLECULAR_ARMS_VS_ERMS_DN	21	**4.9509e-5**	**1.6655e-1**	**7.9309e-3**	2.5022	9.5914	24	0.0247	6.19%	7	**1.0992e-7**	**3.6978e-4**	**5.2826e-5**	3.8898	[illegible]

Name	ID	Rank	P-Value	P-Value	*Val*	Enrichment	Expected	Region Hits	Fraction	Set Coverage	Rank	P-Value	P-Value	*Val*	Enrichment	Ex
**Genes up-regulated in SaOS-2 cells (osteosarcoma) upon knockdown of YY1 [GeneID=7528] by RNAi.**	DE_YY1_TARGETS_UP	22	**6.0863e-5**	**2.0474e-1**	**9.3065e-3**	9.1607	0.6550	6	0.0017	1.55%	62	**4.0794e-4**	**1.0000**	**2.2134e-2**	6.0083	[illegible]
**Genes down-regulated in HCT8/S11 cells (colon cancer) engineered to stably express NTN1 [GeneID=1630] off a plasmid vector.**	RODRIGUES_NTN1_TARGETS_DN	23	**9.1648e-5**	**3.0830e-1**	**1.3405e-2**	2.9553	5.7523	17	0.0148	4.38%	63	**4.1338e-4**	**1.0000**	**2.2073e-2**	2.8575	[illegible]
**Genes with DNA sequences bound by RARA and RARG [GeneID=5914, 5916] in ES cells.**	DELACROIX_RAR_BOUND_ES	25	**1.1679e-4**	**3.9287e-1**	**1.5715e-2**	2.0883	14.8446	31	0.0383	7.99%	50	**2.6753e-4**	**8.9995e-1**	**1.7999e-2**	2.0579	[illegible]
**Genes repressed in the time interval between two pulses of EGF [GeneID =1950] in 184A1 cells (mammary epithelium).**	ZWANG_EGF_INTERVAL_DN	26	**1.2214e-4**	**4.1088e-1**	**1.5803e-2**	2.5347	8.2849	21	0.0214	5.41%	27	**4.1549e-5**	**1.3977e-1**	**5.1767e-3**	2.9579	[illegible]

The test set of 388 genomic regions picked 563 (3%) of all 18,041 genes.
MSigDB Perturbation has 3,364 terms covering 17,091 (95%) of all 18,041 genes, and 358,104 term - gene associations.
3,364 ontology terms (100%) were tested using an annotation count range of [1, Inf].

MSigDB Predicted Promoter Motifs (20+ terms)

Global controls

Table controls: Export ▼ Shown top rows in this table: 20 Set Term annotation count: Min: 1 Max: Inf Set Visualize this table: NEW [select one] ▼

Term Name	Term ID	Binom Rank	Binom Raw P-Value	Binom Bonferroni P-Value	*Binom FDR Q-Val*	Binom Fold Enrichment	Binom Expected	Binom Observed Region Hits	Binom Genome Fraction	Binom Region Set Coverage	Hyper Rank	Hyper Raw P-Value	Hyper Bonferroni P-Value	*Hyper FDR Q-Val*	Hyper Fold Enrichment	Hyper Expected	Hyper Observed Gene Hits	Hyper Total Genes	G C
**Motif CANCCNNWGGGTGDGG (no known TF)**	V$CACCCBINDINGFACTOR_Q6	2	**1.1429e-7**	**7.0285e-5**	**3.5143e-5**	2.7923	12.1763	34	0.0314	8.76%	9	**1.1461e-8**	**7.0488e-6**	**7.8320e-7**	3.4643	8.0825	28	259	
**Motif GGCGSG matches E2F<br>TFDP1: transcription factor Dp-1**	V$E2F_Q2	3	**6.5969e-7**	**4.0571e-4**	**1.3524e-4**	3.4821	6.3180	22	0.0163	5.67%	10	**5.9458e-8**	**3.6567e-5**	**3.6567e-6**	4.0295	5.2115	21	167	
**Motif GRGGSTGGG (no known TF)**	V$CACBINDINGPROTEIN_Q6	4	**2.5596e-6**	**1.5741e-3**	**3.9354e-4**	3.0083	7.9779	24	0.0206	6.19%	73	**1.2927e-4**	**7.9502e-2**	**1.0891e-3**	2.6243	7.2400	19	232	
**Motif NGNGGGGA (no known TF)**	V$MZF1_01	5	**3.4831e-6**	**2.1421e-3**	**4.2842e-4**	2.7356	9.8700	27	0.0254	6.96%	40	**1.2302e-5**	**7.5659e-3**	**1.8915e-4**	2.9131	7.2087	21	231	
**Motif NNGKNTGTGGTTWNC matches RUNX1: runt-related transcription factor 1 (acute myeloid leukemia 1; aml1 oncogene)**	V$AML_Q6	6	**5.7329e-6**	**3.5258e-3**	**5.8763e-4**	2.5503	11.3711	29	0.0293	7.47%	33	**6.6255e-6**	**4.0747e-3**	**1.2347e-4**	2.8567	8.0513	23	258	
**Motif GCHCDAMCCAG matches TFCP2: transcription factor CP2**	V$CP2_01	7	**5.8144e-6**	**3.5758e-3**	**5.1083e-4**	2.6013	10.7639	28	0.0277	7.22%	31	**5.8231e-6**	**3.5812e-3**	**1.1552e-4**	2.8790	7.9889	23	256	
**Motif NNGTTGTTTACNTN matches MLLT7: myeloid/lymphoid or mixed-lineage leukemia (trithorax homolog, Drosophila); translocated to, 7**	V$FOXO4_02	8	**7.8915e-6**	**4.8533e-3**	**6.0666e-4**	2.2352	16.1061	36	0.0415	9.28%	5	**1.4785e-9**	**9.0927e-7**	**1.8185e-7**	3.6877	7.8641	29	252	
**Motif NNTTTCCN matches STAT1: signal transducer and activator of transcription 1, 91kDa**	V$STAT1_03	9	**1.0086e-5**	**6.2030e-3**	**6.8922e-4**	2.6986	9.2640	25	0.0239	6.44%	103	**5.8812e-4**	**3.6169e-1**	**3.5116e-3**	2.3934	7.5208	18	241	
**Motif GCGSCMNTTT (no known TF)**	GCGSCMNTTT_UNKNOWN	10	**1.8928e-5**	**1.1641e-2**	**1.1641e-3**	4.5524	2.6360	12	0.0068	3.09%	179	**4.7865e-3**	**1.0000**	**1.6445e-2**	3.3479	2.0908	7	67	
**Motif CNGNRNCAGGTGNNGNAN matches MYOD1: myogenic differentiation 1**	V$MYOD_Q6_01	11	**2.0476e-5**	**1.2592e-2**	**1.1448e-3**	2.5269	10.2891	26	0.0265	6.70%	61	**7.2169e-5**	**4.4384e-2**	**7.2761e-4**	2.6593	7.5208	20	241	
**Motif ATGCCCATATATGGWNNT matches SRF: serum response factor (c-fos serum response element-binding transcription factor)**	V$SRF_01	14	**9.1845e-5**	**5.6484e-2**	**4.0346e-3**	5.7981	1.3798	8	0.0036	2.06%	165	**4.0255e-3**	**1.0000**	**1.5004e-2**	3.9238	1.5291	6	49	
**Motif CACSCCA matches SREBF1: sterol regulatory element binding transcription factor 1**	V$SREBP1_Q6	15	**1.0584e-4**	**6.5093e-2**	**4.3396e-3**	2.4345	9.4477	23	0.0243	5.93%	79	**1.6155e-4**	**9.9352e-2**	**1.2576e-3**	2.5798	7.3648	19	236	

Name	ID	Rank	P-Value	Bonferroni P-Value	FDR Q Val	Fold Enrichment	Expected	Region Hits	Genome Fraction	Set Coverage	Rank	Raw P-Value	Bonferroni P-Value	FDR Q Val	Fold Enrichment	Expected	Gene Hits	Total Genes	C
**Motif TATAAATW matches TBP: TATA box binding protein**	V$TBP_01	16	**1.1421e-4**	**7.0239e-2**	**4.3899e-3**	2.2304	12.1054	27	0.0312	6.96%	70	**1.2215e-4**	**7.5123e-2**	**1.0732e-3**	2.6357	7.2087	19	231	
**Motif TGTGGTTW matches CBFA2T2: core-binding factor, runt domain, alpha subunit 2; translocated to, 2<br>CBFA2T3: core-binding factor, runt domain, alpha subunit 2; translocated to, 3**	V$COREBINDINGFACTOR_Q6	17	**1.2123e-4**	**7.4556e-2**	**4.3857e-3**	2.2221	12.1505	27	0.0313	6.96%	107	**7.5573e-4**	**4.6477e-1**	**4.3437e-3**	2.2803	8.3322	19	267	
**Motif TGACCTTTGNCCY matches PPARA: peroxisome proliferative activated receptor, alpha**	V$PPAR_DR1_Q2	18	**1.6267e-4**	**1.0004e-1**	**5.5580e-3**	2.4813	8.4632	21	0.0218	5.41%	239	**1.1379e-2**	**1.0000**	**2.9281e-2**	1.9382	7.7393	15	248	
**Motif NNNTTCCN matches STAT3: signal transducer and activator of transcription 3 (acute-phase response factor)**	V$STAT3_02	19	**1.7277e-4**	**1.0625e-1**	**5.5922e-3**	2.6153	7.2649	19	0.0187	4.90%	83	**1.9500e-4**	**1.1993e-1**	**1.4449e-3**	3.0728	4.5562	14	146	
**Motif NNNGGNCNCAGCTGCGNCCCNN matches NHLH1: nescient helix loop helix 1**	V$HEN1_01	20	**1.8748e-4**	**1.1530e-1**	**5.7649e-3**	2.3942	9.1888	22	0.0237	5.67%	42	**1.3799e-5**	**8.4861e-3**	**2.0205e-4**	3.0906	6.1477	19	197	
**Motif NNNRRCCAATSRGNNN (no known TF)**	V$NFY_01	22	**1.8885e-4**	**1.1614e-1**	**5.2792e-3**	2.2877	10.4907	24	0.0270	6.19%	138	**1.9214e-3**	**1.0000**	**8.5628e-3**	2.2145	7.6768	17	246	
**Motif CCCCAAACMMCCCC matches RREB1: ras responsive element binding protein 1**	V$RREB1_01	23	**2.3667e-4**	**1.4555e-1**	**6.3285e-3**	2.3537	9.3471	22	0.0241	5.67%	60	**5.7951e-5**	**3.5640e-2**	**5.9400e-4**	2.8840	6.2413	18	200	
**Motif NNNTGGGAWNNC (no known TF)**	V$IK2_01	24	**2.3709e-4**	**1.4581e-1**	**6.0755e-3**	2.0326	14.7596	30	0.0380	7.73%	24	**2.2839e-6**	**1.4046e-3**	**5.8524e-5**	2.9579	8.1137	24	260	

The test set of 388 genomic regions picked 563 (3%) of all 18,041 genes.
MSigDB Predicted Promoter Motifs has 615 terms covering 11,991 (66%) of all 18,041 genes, and 158,271 term - gene associations.
615 ontology terms (100%) were tested using an annotation count range of [1, Inf].

MSigDB miRNA Motifs (no terms)

Global controls

Table controls: Export ▾ Shown top rows in this table: 20 Set Term annotation count: Min: 1 Max: Inf Set Visualize this table: [select one] ▾

Term Name	Term ID	Binom Rank	Binom Raw P-Value	Binom Bonferroni P-Value	*Binom FDR Q-Val*	Binom Fold Enrichment	Binom Expected	Binom Observed Region Hits	Binom Genome Fraction	Binom Region Set Coverage	Hyper Rank	Hyper Raw P-Value	Hyper Bonferroni P-Value	*Hyper FDR Q-Val*	Hyper Fold Enrichment	Hyper Expected	Hyper Observed Gene Hits	Hyper Total Genes	Hyper Gene Set Coverage	Hyper Term Gene Coverage

No results meet your chosen criteria.

The test set of 388 genomic regions picked 563 (3%) of all 18,041 genes.
MSigDB miRNA Motifs has 221 terms covering 7,064 (39%) of all 18,041 genes, and 32,882 term - gene associations.
221 ontology terms (100%) were tested using an annotation count range of [1, Inf].

InterPro (6 terms)

Global controls

Table controls: Export ▾ Shown top rows in this table: 20 Set Term annotation count: Min: 1 Max: Inf Set Visualize this table: [select one] ▾

Term Name	Term ID	Binom Rank	Binom Raw P-Value	Binom Bonferroni P-Value	*Binom FDR Q-Val*	Binom Fold Enrichment	Binom Expected	Binom Observed Region Hits	Binom Genome Fraction	Binom Region Set Coverage	Hyper Rank	Hyper Raw P-Value	Hyper Bonferroni P-Value	*Hyper FDR Q-Val*	Hyper Fold Enrichment	Hyper Expected	Hyper Observed Gene Hits	Hyper Total Genes	Hyper Gene Set Coverage	Hyper Term Gene Coverage
**Homeobox protein, antennapedia type, conserved site**	IPR001827	1	**4.4089e-27**	**4.1541e-23**	**4.1541e-23**	239.5964	0.0543	13	0.0001	3.35%	5	**1.1286e-10**	**1.0634e-6**	**2.1268e-7**	16.0222	0.6241	10	20	1.78%	50.00%
**Homeobox protein, antennapedia type**	IPR017995	2	**1.3625e-19**	**1.2838e-15**	**6.4189e-16**	167.1899	0.0598	10	0.0002	2.58%	6	**1.9197e-5**	**1.8088e-1**	**3.0146e-2**	13.3518	0.3745	5	12	0.89%	41.67%

Name	ID	Rank	P-Value	P-Value	Val	Enrichment	Expected	Region Hits	Fraction	Set Coverage	Rank	P-Value	P-Value	Val	Enrichment	Expected	Gene Hits	Genes	Coverage	Gene Coverage
**Homeodomain, metazoa**	IPR020479	3	**3.5144e-14**	**3.3112e-10**	**1.1037e-10**	8.1590	2.8190	23	0.0073	5.93%	4	**5.6631e-12**	**5.3358e-8**	**1.3339e-8**	6.9662	2.8710	20	92	3.55%	21.74%
**Homeobox, conserved site**	IPR017970	4	**3.7423e-11**	**3.5260e-7**	**8.8149e-8**	3.8671	8.7921	34	0.0227	8.76%	1	**2.6966e-14**	**2.5408e-10**	**2.5408e-10**	5.2839	5.8669	31	188	5.51%	16.49%
**Homeobox domain**	IPR001356	5	**2.2310e-9**	**2.1020e-5**	**4.2041e-6**	3.0338	12.5253	38	0.0323	9.79%	2	**3.8739e-14**	**3.6500e-10**	**1.8250e-10**	4.6154	7.5832	35	243	6.22%	14.40%
**Homeodomain-like**	IPR009057	6	**2.4742e-8**	**2.3312e-4**	**3.8853e-5**	2.6000	16.1537	42	0.0416	10.82%	3	**6.6471e-13**	**6.2629e-9**	**2.0876e-9**	3.8218	10.2046	39	327	6.93%	11.93%

The test set of 388 genomic regions picked 563 (3%) of all 18,041 genes.
InterPro has 9,422 terms covering 17,343 (96%) of all 18,041 genes, and 60,477 term - gene associations.
9,422 ontology terms (100%) were tested using an annotation count range of [1, Inf].

TreeFam (no terms)

Global controls

Table controls: Export ▾ Shown top rows in this table: 20 Set Term annotation count: Min: 1 Max: Inf Set Visualize this table: NEW [select one] ▾

Term Name	Term ID	Binom Rank	Binom Raw P-Value	Binom Bonferroni P-Value	*Binom FDR Q-Val*	Binom Fold Enrichment	Binom Expected	Binom Observed Region Hits	Binom Genome Fraction	Binom Region Set Coverage	Hyper Rank	Hyper Raw P-Value	Hyper Bonferroni P-Value	*Hyper FDR Q-Val*	Hyper Fold Enrichment	Hyper Expected	Hyper Observed Gene Hits	Hyper Total Genes	Hyper Gene Set Coverage	Hyper Term Gene Coverage

No results meet your chosen criteria.

The test set of 388 genomic regions picked 563 (3%) of all 18,041 genes.
TreeFam has 8,126 terms covering 13,550 (75%) of all 18,041 genes, and 13,551 term - gene associations.
8,126 ontology terms (100%) were tested using an annotation count range of [1, Inf].

HGNC Gene Families (1 term)

Global controls

Table controls: Export ▾ Shown top rows in this table: 20 Set Term annotation count: Min: 1 Max: Inf Set Visualize this table: NEW [select one] ▾

Term Name	Term ID	Binom Rank	Binom Raw P-Value	Binom Bonferroni P-Value	*Binom FDR Q-Val*	Binom Fold Enrichment	Binom Expected	Binom Observed Region Hits	Binom Genome Fraction	Binom Region Set Coverage	Hyper Rank	Hyper Raw P-Value	Hyper Bonferroni P-Value	*Hyper FDR Q-Val*	Hyper Fold Enrichment	Hyper Expected	Hyper Observed Gene Hits	Hyper Total Genes	Hyper Gene Set Coverage	Hyper Term Gene Coverage
**HOXL**	HOXL	1	**1.5139e-18**	**7.2366e-16**	**7.2366e-16**	25.0014	0.6800	17	0.0018	4.38%	1	**3.3259e-11**	**1.5898e-8**	**1.5898e-8**	9.2436	1.6227	15	52	2.66%	28.85%

The test set of 388 genomic regions picked 563 (3%) of all 18,041 genes.
HGNC Gene Families has 478 terms covering 7,310 (41%) of all 18,041 genes, and 7,310 term - gene associations.
478 ontology terms (100%) were tested using an annotation count range of [1, Inf].

Ensembl Genes (no terms)

Global controls

Table controls: Export ▾ Shown top rows in this table: 20 Set Term annotation count: Min: 1 Max: Inf Set Visualize this table: NEW [select one] ▾

Term Name	Term ID	Binom Rank	Binom Raw P-Value	Binom Bonferroni P-Value	*Binom FDR Q-Val*	Binom Fold Enrichment	Binom Expected	Binom Observed Region Hits	Binom Genome Fraction	Binom Region Set Coverage	Hyper Rank	Hyper Raw P-Value	Hyper Bonferroni P-Value	*Hyper FDR Q-Val*	Hyper Fold Enrichment	Hyper Expected	Hyper Observed Gene Hits	Hyper Total Genes	Hyper Gene Set Coverage	Hyper Term Gene Coverage

No results meet your chosen criteria.

18,041 ontology terms (100%) were tested using an annotation count range of [1, Inf].

MSigDB Oncogenic Signatures (2 terms)

Global controls

Table controls: Export ▼ Shown top rows in this table: 20 Set Term annotation count: Min: 1 Max: Inf Set Visualize this table: [select one] ▼

Term Name	Term ID	Binom Rank	Binom Raw P-Value	Binom Bonferroni P-Value	*Binom FDR Q-Val*	Binom Fold Enrichment	Binom Expected	Binom Observed Region Hits	Binom Genome Fraction	Binom Region Set Coverage	Hyper Rank	Hyper Raw P-Value	Hyper Bonferroni P-Value	*Hyper FDR Q-Val*	Hyper Fold Enrichment	Hyper Expected	Hyper Observed Gene Hits	Hyper Total Genes	Hyper Gene Set Coverage	Hy Te Ge Cove
**Genes up-regulated in small intenstine in PRKCA [Gene ID=5578] knockout mice.**	PKCA_DN.V1_UP	1	**2.7178e-6**	**5.0823e-4**	**5.0823e-4**	3.9160	4.3412	17	0.0112	4.38%	6	**1.8345e-3**	**3.4305e-1**	**5.7175e-2**	2.5557	5.0867	13	163	2.31%	7.9
**Genes up-regulated in Sez-4 cells (T lymphocyte) that were first starved of IL2 [Gene ID=3558] and then stimulated with IL2 [Gene ID=3558].**	IL2_UP.V1_UP	2	**1.9864e-4**	**3.7145e-2**	**1.8573e-2**	2.5861	7.3469	19	0.0189	4.90%	3	**5.0627e-4**	**9.4672e-2**	**3.1557e-2**	2.6853	5.5860	15	179	2.66%	8.3

The test set of 388 genomic regions picked 563 (3%) of all 18,041 genes.
MSigDB Oncogenic Signatures has 187 terms covering 10,315 (57%) of all 18,041 genes, and 30,004 term - gene associations.
187 ontology terms (100%) were tested using an annotation count range of [1, Inf].

MSigDB Immunologic Signatures (no terms)

Global controls

Table controls: Export ▼ Shown top rows in this table: 20 Set Term annotation count: Min: 1 Max: Inf Set Visualize this table: [select one] ▼

Term Name	Term ID	Binom Rank	Binom Raw P-Value	Binom Bonferroni P-Value	*Binom FDR Q-Val*	Binom Fold Enrichment	Binom Expected	Binom Observed Region Hits	Binom Genome Fraction	Binom Region Set Coverage	Hyper Rank	Hyper Raw P-Value	Hyper Bonferroni P-Value	*Hyper FDR Q-Val*	Hyper Fold Enrichment	Hyper Expected	Hyper Observed Gene Hits	Hyper Total Genes	Hyper Gene Set Coverage	Hyper Term Gene Coverage

No results meet your chosen criteria.

The test set of 388 genomic regions picked 563 (3%) of all 18,041 genes.
MSigDB Immunologic Signatures has 1,910 terms covering 16,609 (92%) of all 18,041 genes, and 363,333 term - gene associations.
1,910 ontology terms (100%) were tested using an annotation count range of [1, Inf].

STANFORD SCHOOL of MEDICINE Bejerano Lab STANFORD ENGINEERING

Copyright © 2010-2017. The Board of Trustees of Leland Stanford Junior University. All rights reserved.

Terms of Use About Us Contact Us
