# Supplementary material for: Growth disrupting mutations in epigenetic regulatory molecules are associated with abnormalities of epigenetic aging
Source: Genome Res. 2019 Jul;29(7):1057–66. doi: 10.1101/gr.243584.118 (PMC6633263; doi:10.1101/gr.243584.118)
Supplement: Supplemental Material [file supp_gr.243584.118_Supplemental_Material.pdf]

Supplemental Material

Growth disrupting mutations in epigenetic regulatory molecules are associated with abnormalities of epigenetic aging

Aaron R Jeffries, Reza Maroofian, Claire G. Salter, Barry A. Chioza, Harold E. Cross, Michael A. Patton, Emma Dempster, I. Karen Temple, Deborah Mackay, Faisal I. Rezwan, Lise Aksglæde, Diana Baralle, Tabib Dabir, Matthew Frank Hunter, Arveen Kamath, Ajith Kumar, Ruth Newbury-Ecob, Angelo Selicorni, Amanda Springer, Lionel van Maldergem, Vinod Varghese, Naomi Yachelevich, Katrina Tatton-Brown, Jonathan Mill, Andrew H. Crosby and Emma Baple

Table of Contents:

- *Supplemental Figures S1 - S8 (pages 2 - 9)*
- *Supplemental Tables S2, S5 -S7 (pages 10 -13)*

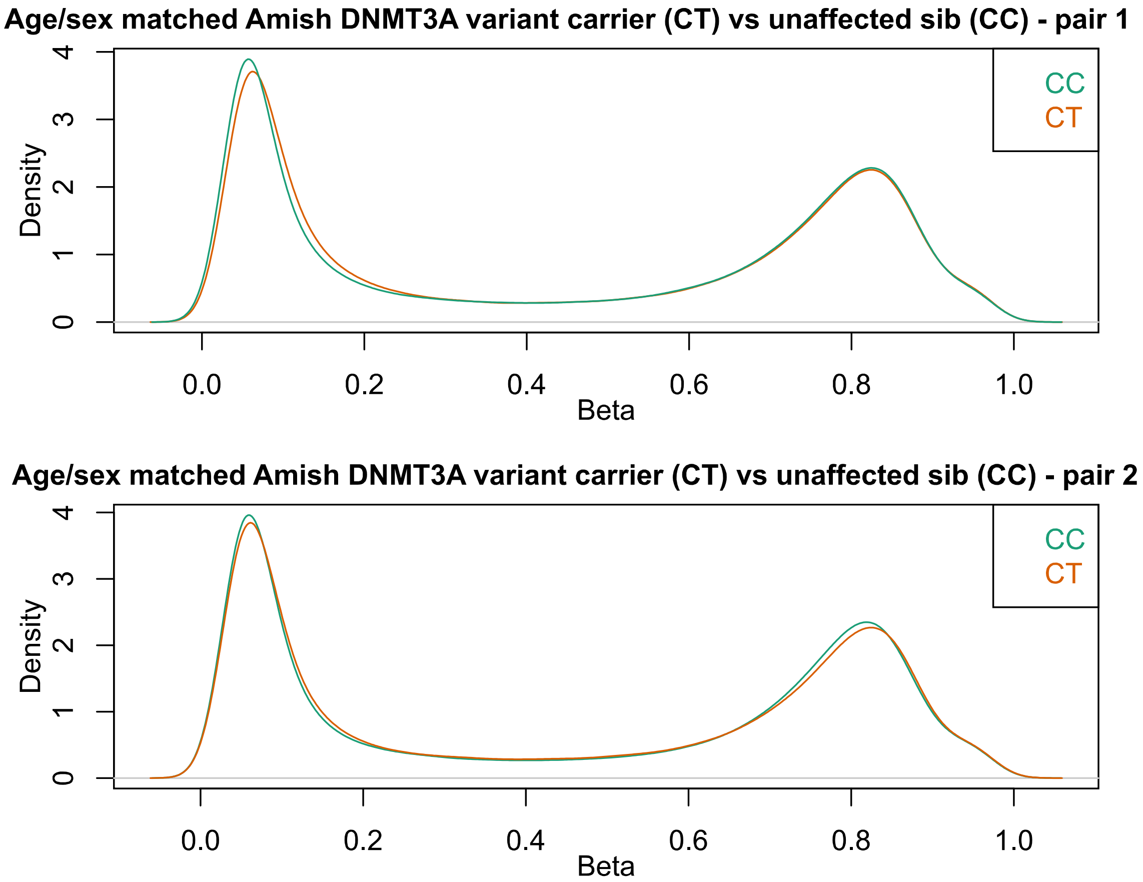

Summary statistics and Wilcoxon Rank Sum test based on autosomal CpGs (n=414,172)

Pair 1 (19y and 20y female)

	CC	CT	difference
**Mean Beta**	0.46302	0.459845	-0.003175
**Median Beta**	0.536653	0.522289	-0.014364

Wicoxon Rank Sum p-value = 0.2417

Pair 2 (24y and 25y female)

	CC	CT	difference
**Mean Beta**	0.461421	0.458581	-0.00284
**Median Beta**	0.540997	0.517324	-0.023673

Wicoxon Rank Sum p-value = 0.1409

Supplemental Figure S1 – Global methylation comparison inferred from Illumina 450k methylation array.
Density plots of methylation beta values for two pairs of Amish *DNMT3A* wildtype (CC) and c.2312G>A; p.(Arg771Gln) variant carriers (CT) which are age and sex matched. Summary statistics are also shown together with a Wilcoxon Rank Sum p-value for comparison between individuals.

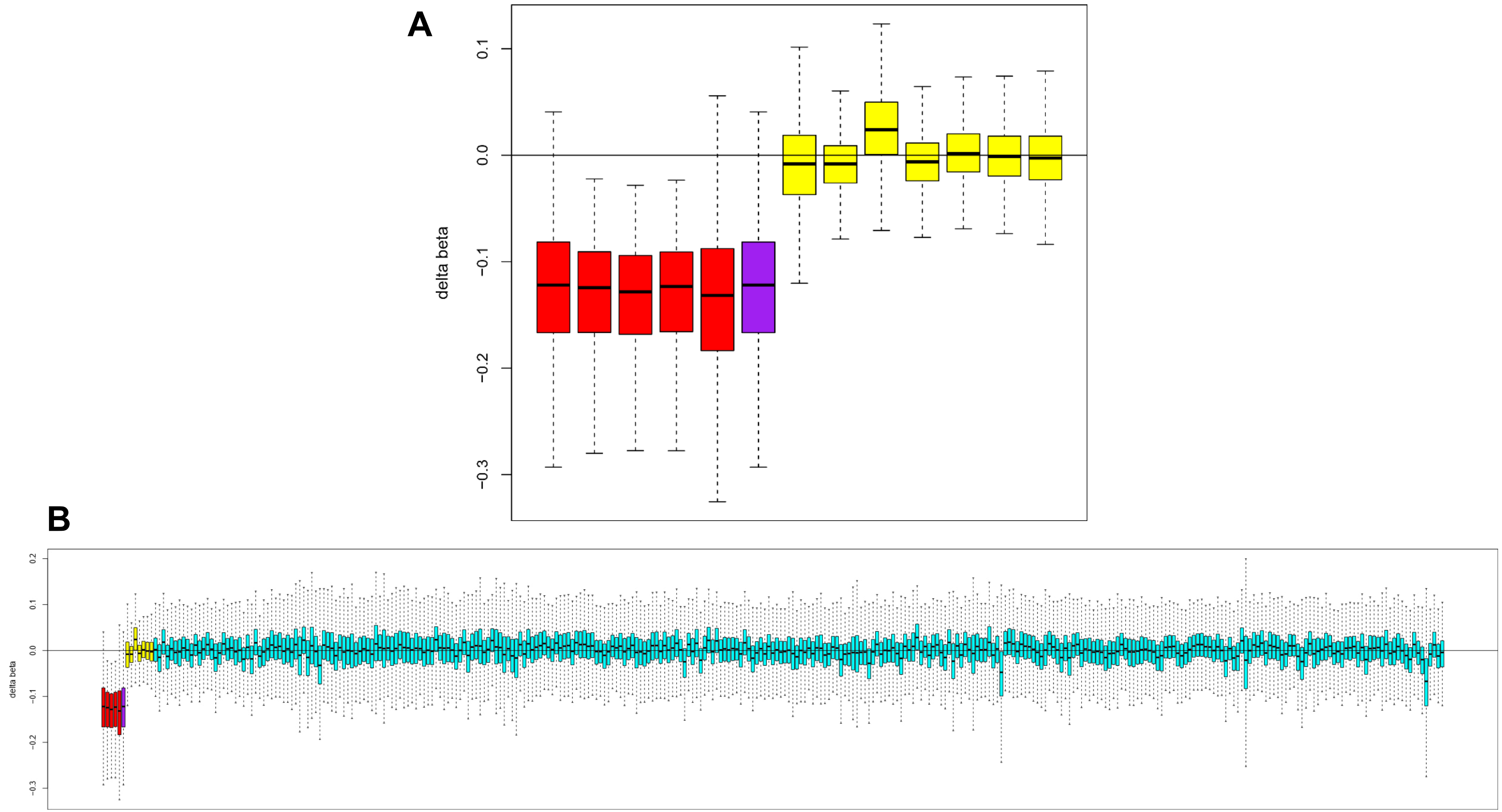

Supplemental Figure S2 – Boxplot illustrating the magnitude of DNA methylation changes observed *DNMT3A* c.2312G>A; p.(Arg771Gln) carriers vs wildtype individuals. (**A**) Boxplot showing the distinct difference in DNA methylation across *DNMT3A* c.2312G>A; p.(Arg771Gln) DMPs, shown as a delta beta value, for the Amish c.2312G>A; p.(Arg771Gln) heterozygotes (red) and a mosaic individual (purple) compared to Amish wildtype individuals (yellow). (**B**) Extended boxplot as above but with additional control data (cyan) derived from 322 individuals in a general population cohort.

A

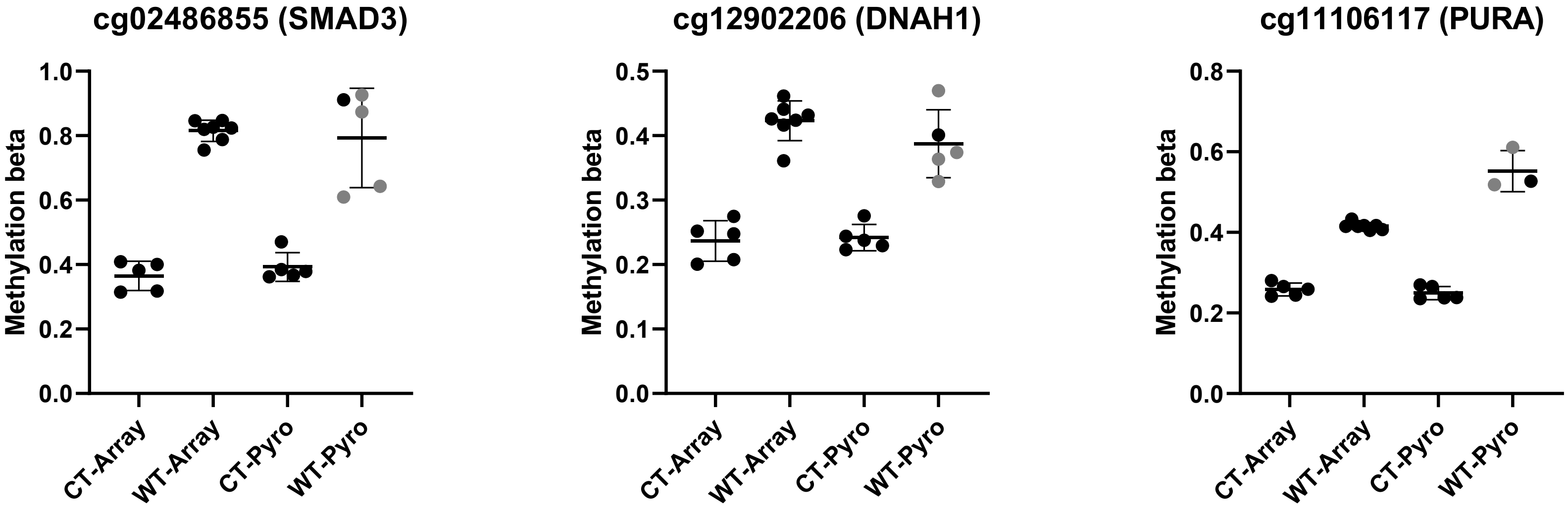

B

	T-test p-value		delta beta	
	Array	Pyro	Array	Pyro
cg02486855	1.04E-09	2.59E-04	-0.45	-0.40
cg12092206	6.47E-07	2.17E-04	-0.19	-0.15
cg11106117	4.09E-10	7.11E-06	-0.16	-0.30

C

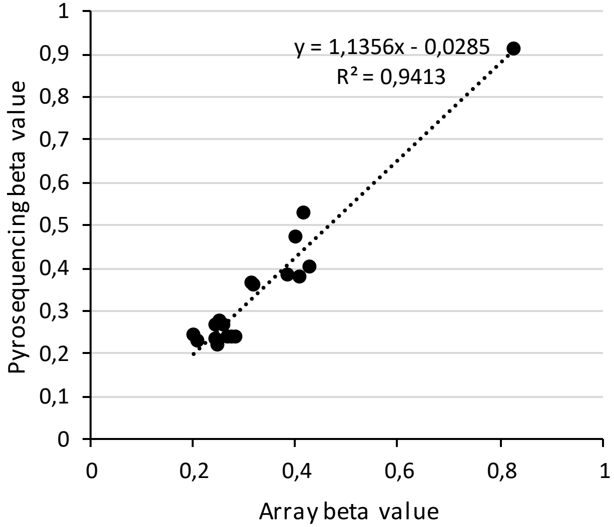

Supplemental Figure S3 – Pyrosequencing validation of specific DMPs associated with the *DNMT3A* c.2312G>A; p.(Arg771Gln) variant. (**A**) Examination of *DNMT3A* c.2312G>A; p.(Arg771Gln) heterozygous carriers (labelled CT) vs wildtype (labelled WT) comparing DNA methylation estimates derived from the Illumina 450K methylation array (Array) and bisulfite-pyrosequencing (Pyro). Unrelated wildtype individuals were also included (grey). (**B**) T-test results show that both array and pyrosequencing results are fully concordant. (**C**) Correlation of Pyrosequencing vs Array measurements for Amish samples measured on both platforms.

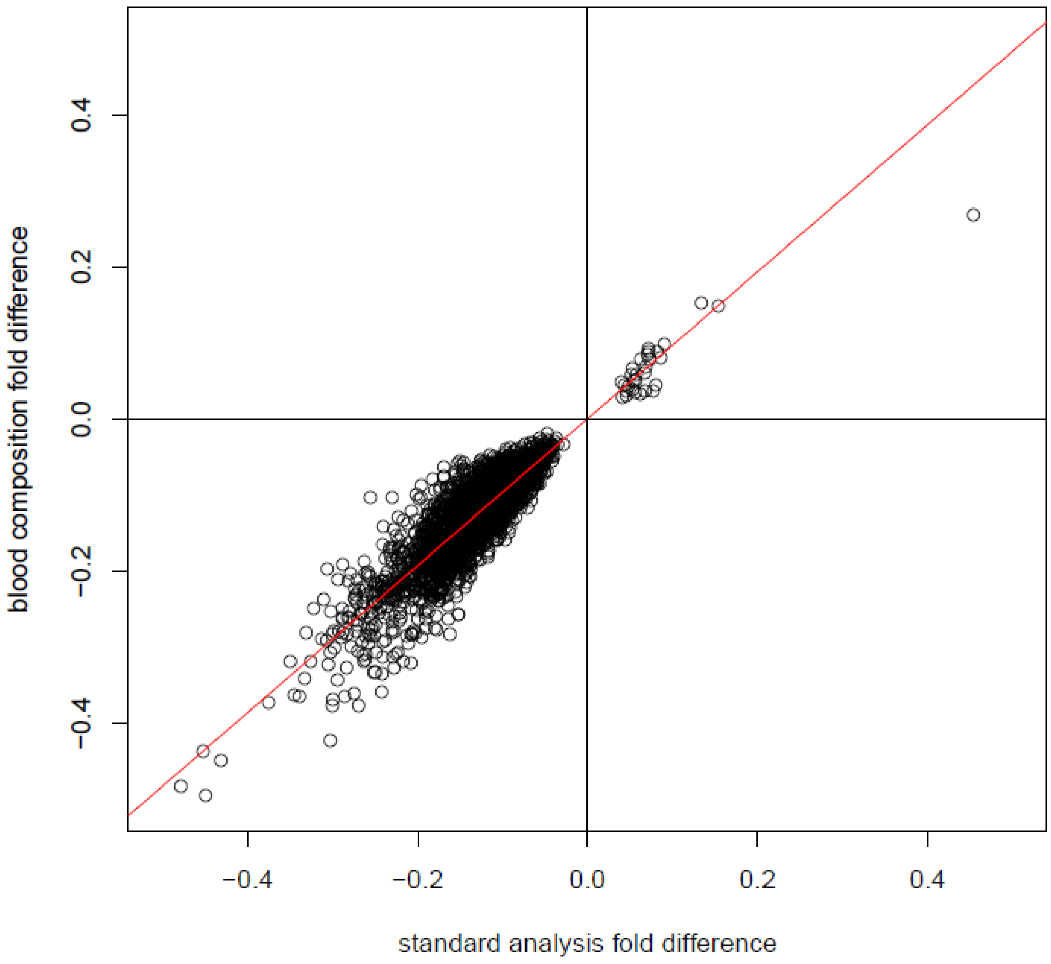

Supplemental Figure S4 – Scatterplot highlighting the negligible effect of including derived blood cell proportions as a covariate in our analysis of differential DNA methylation associated with the DNMT3A c.2312G>A p.(Arg771Gln) pathogenic variant.

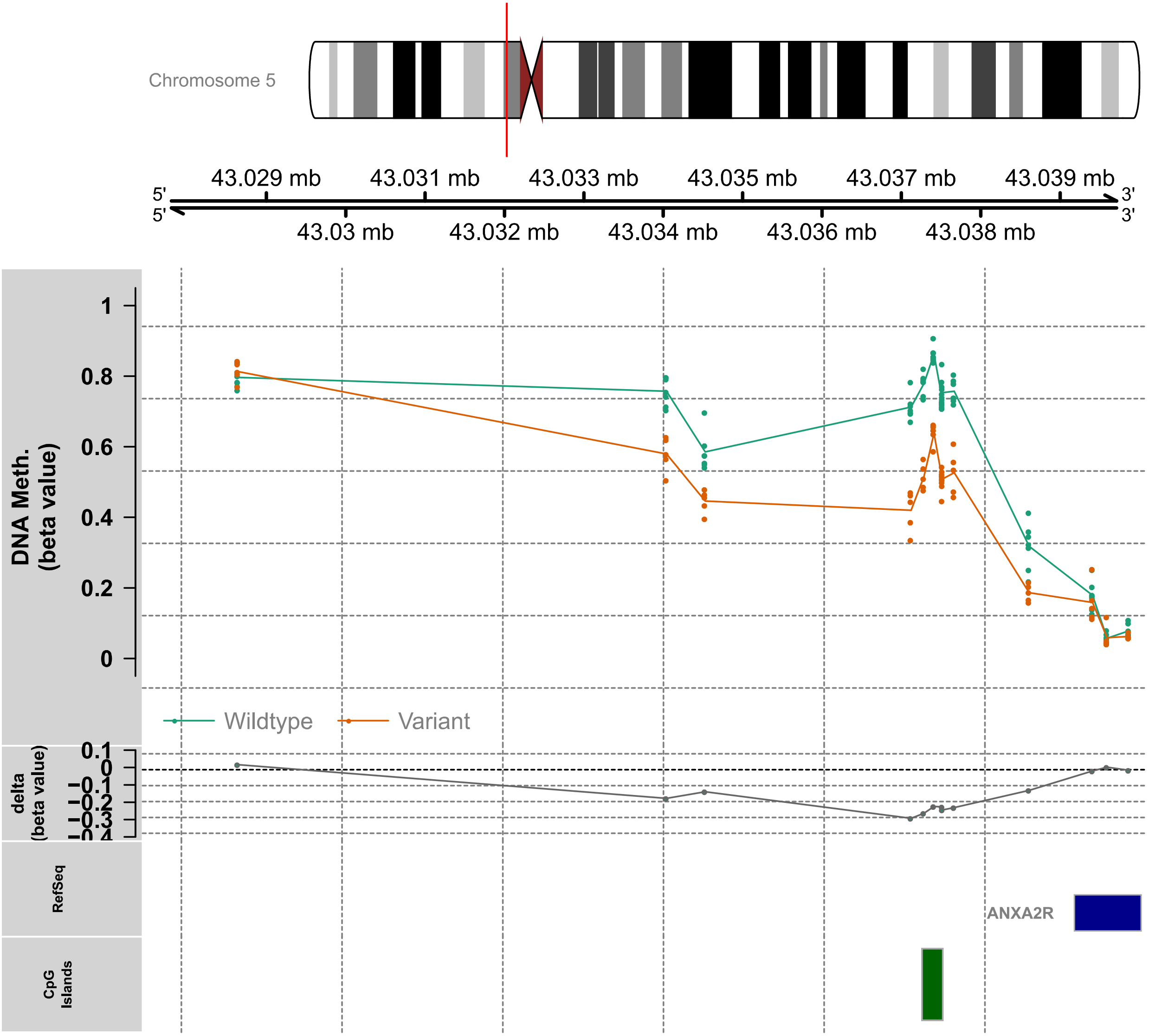

Supplemental Figure S5 – Example of the top ranked DMR located immediately upstream of the gene *ANXA2R*. Methylation beta values for Amish *DNMT3A* c.2312G>A; p.(Arg771Gln) heterozygous carriers (orange) are shown together with wildtype family members (green) and their delta(beta) value difference (change in methylation) in the lower row.

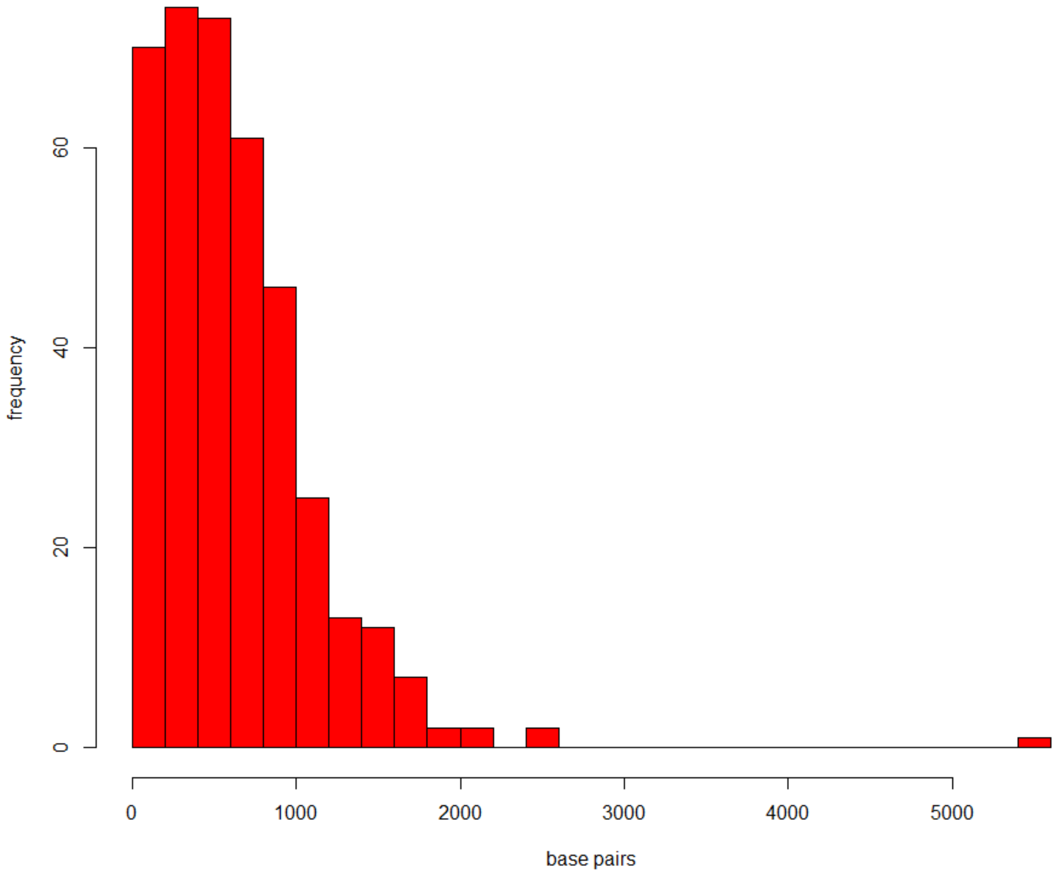

Supplemental Figure S6 – Size distribution of differentially methylated regions. Size distribution (in base pairs) of differentially methylated regions (DMRs) in Amish *DNMT3A* c.2312G>A; p.(Arg771Gln) heterozygotes vs wildtype family members, detected on the Illumina 450k methylation array.

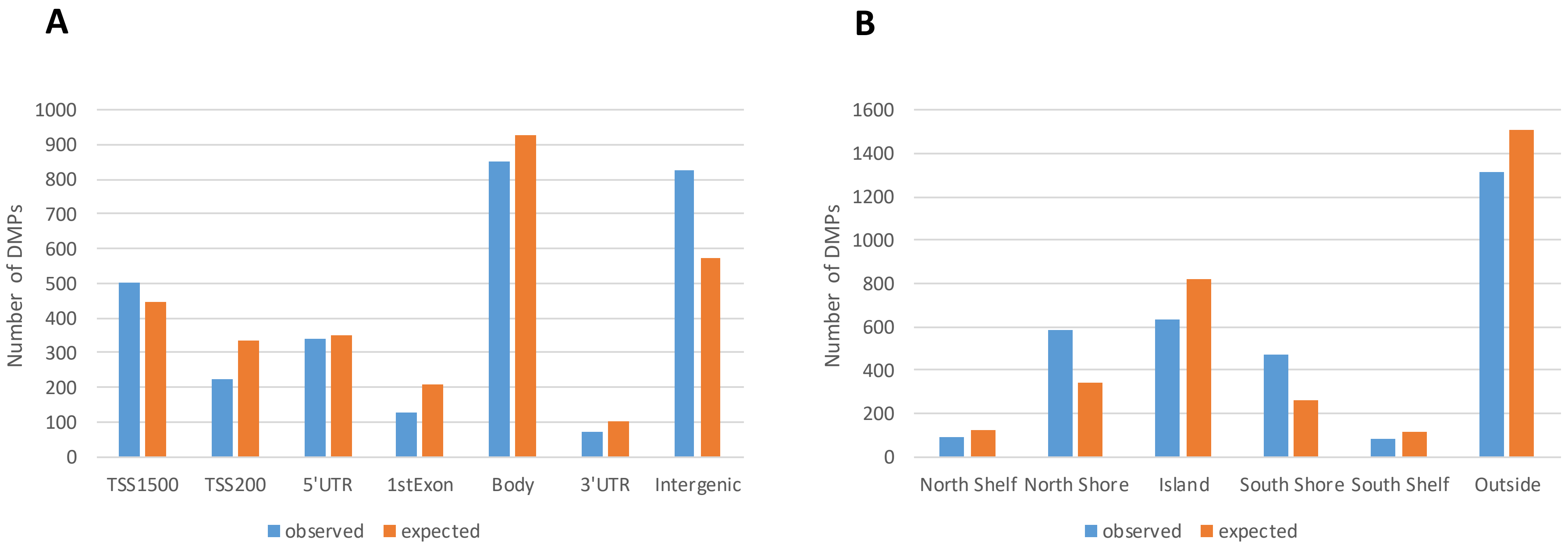

Supplemental Figure S7 – Annotation of differentially methylated positions relative to transcripts and CpG islands. Bar chart showing the relative enrichment of differentially methylated positions (DMPs) annotated to regions around the transcript (**A**) and CpG Islands (**B**). The bars indicate the observed (orange) and expected number (blue) of DMPs found in Amish *DNMT3A* c.2312G>A; p.(Arg771Gln) variant carriers.

Annotation abbreviations (from Illumina supplied 450k methylation array annotation): *TSS1500*; 1500bp upstream of the transcriptional start site, *TSS200*; 200bp upstream of the transcriptional start site/the promoter, *5'UTR*; 5' Untranslated region, *3'UTR*; 3' Untranslated region, *1stExon*; First exon of the transcript, *Body*; probes which lie within other exons or introns of the transcript. *Island*; CpG Island, *Shore*; region upto 2kb from CpG Island, *Shelf*; 2-4kb from CpG Island, *North*; upstream, *South*; downstream, *Outside*; probes outside of the CpG Island defined regions.

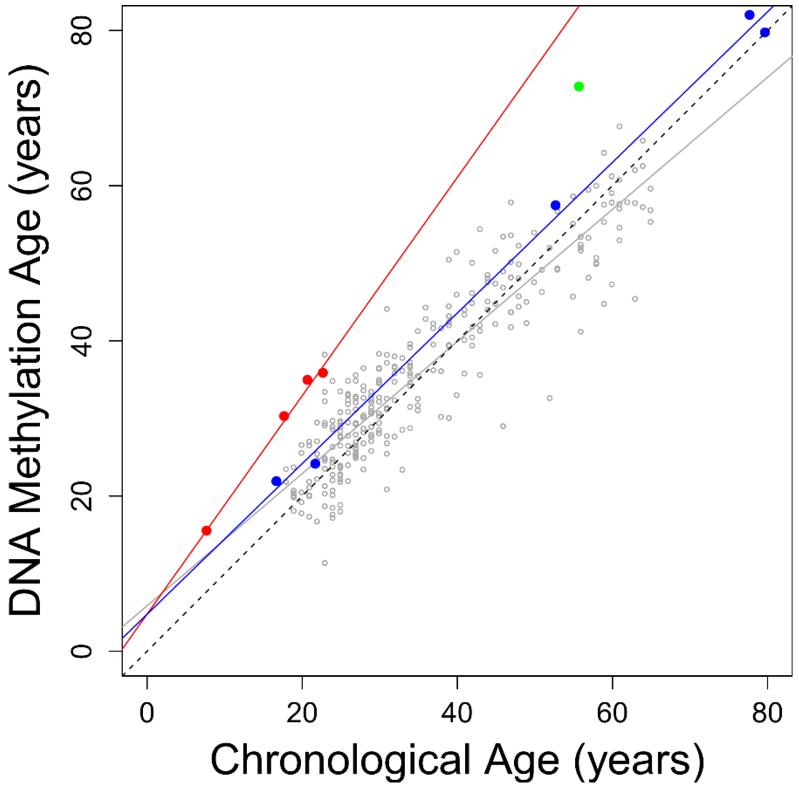

Supplemental Figure S8 – Scatterplot showing deviations in epigenetic age estimates compared to actual chronological age in *DNMT3A* c.2312G>A p.(Arg771Gln) pathogenic variant carriers vs wildtype (Amish and anonymous controls).
Scatter plot comparing 'DNA methylation age' derived from the Illumina 450K data (y-axis) and chronological age (x-axis) in *DNMT3A* c.2312G>A p.(Arg771Gln) heterozygotes (red) vs wildtype family members (blue) and a control cohort of 322 individuals (grey). Green indicates the mosaic individual. The line of best fit is also shown for each group.

ID	Genotype	Age	CD8T	CD4T	NK	Bcell	Mono	Gran
I.1	CC	78	0.235809	0.038842	0.121331	0.006566	0.079375	0.438575
I.2	CC	80	0.181353	0.224386	0.266975	0.034871	0.082915	0.155131
III.6	CC	19	0.072656	0.146401	0.073649	0.034592	0.092776	0.524193
III.8	CC	24	0.053243	0.099104	0.036907	0.025218	0.120297	0.603879
II.2	CC	53	0.096214	0.123345	0.034925	0.019295	0.136738	0.536551
III.2	CC	-	0.118184	0.132322	0.05267	0.040697	0.071889	0.542375
III.4	CC	-	0.05448	0.152441	0.035462	0.059327	0.073272	0.597367
III.7	CT	20	0.115597	0.175423	0.03418	0.030303	0.067688	0.526625
III.13	CT	10	0.080895	0.146419	0.068219	0.097341	0.104245	0.444545
II.1	Mosaic	58	0.086618	0.080824	0.020623	0.020572	0.104471	0.604476
III.3	CT	25	0.086252	0.13483	0.030652	0.023665	0.100077	0.551573
III.5	CT	23	0.118437	0.158425	0.076498	0.038052	0.089031	0.464135

**Genotype:**	**CT**	**Mean**	0.09756	0.139184	0.046034	0.041987	0.093102	0.518271
		**SD**	0.017934	0.035919	0.024714	0.031664	0.015529	0.065146

**Genotype:***	**CC**	**Mean**	0.078955	0.130723	0.046723	0.035826	0.098995	0.560873
		**SD**	0.028003	0.021061	0.016753	0.015519	0.028781	0.036948

***F*-test p-value**	0.409175	0.325738	0.469955	0.19603	0.259166	0.297722
***T*-test p-value**	**0.246267**	**0.6616**	**0.960154**	**0.706228**	**0.697585**	**0.239121**

* 78 and 80 year old excluded from this group as outliers.

Supplemental Table S2 – A comparison of derived blood cell proportion estimates between *DNMT3A* c.2312G>A p.(Arg771Gln) carriers vs wildtype individuals. Cell composition was deduced from the DNA methylation age calculator and a *t*-test applied to determine any differences between the *DNMT3A* c.2312G>A p.(Arg771Gln) carriers (labelled as CT or mosaic in the genotype column) vs wildtype family members (CC). No significant difference was found in the cell types examined.

Whole gene deletions

Sample ID	Exon	Mutation	Protein change	Inheritance	Sex	Age at sample collection (years)
11D_0326	2Mb deletion	chr5:175,366,008-177,470,488 (hg19)		*De novo*	F	9
11D_0328	1.3 Mb deletion	chr5:175,764,262-177,059,256 (hg19)		*De novo*	F	7
DL151889	2Mb deletion	microdeletion of distal 5q35.2		*De novo*	M	2.2
DL87406	1.9Mb deletion	5q35.2-35.3 (RP11-67P18 to RP11-423H2), FISH BAC RP11-99N22 deleted		*De novo*	M	<1

Intragenic deletions

Sample ID	Exon	Mutation	Protein change	Inheritance	Sex	Age at sample collection (years)
DL38402	5	c.1583delA	p.Lys528Argfs*8	*De novo*	M	19.7
DL50448	5	c.2014-2018delACAGA	p.Thr672Glufs*9	Inherited from father	M	8
DL50450	5	c.2014-2018delACAGA	p.Thr672Glufs*9	*De novo*	M	41
DL50452	5	c.2014-2018delACAGA	p.Thr672Glufs*9	Inherited from father	F	2
11D/6718	5	c.1716delC	p.Cys573Valfs*26	*De novo*	F	10
DL122057	13	c.4843delT	p.Tyr1615Thrfs*27	*De novo*	M	3
11D/6637	15-19	ex15-19 del		*De novo*	M	10

Insertion

Sample ID	Exon	Mutation	Protein change	Inheritance	Sex	Age at sample collection (years)
DL94609	14	4977_4978insG	p.Arg1660Alafs*13	*De novo*	M	20

Nonsense mutations

Sample ID	Exon	Mutation	Protein change	Inheritance	Sex	Age at sample collection (years)
DL159249	22	c.6349C>T	p.Arg2117*	*De novo*	F	12
DL168744	5	c.1492C>T	p.Arg498*	*De novo*	M	2.2
DL89813	5	c.1801A>T	p.Lys601*	*De novo*	M	10.6
DL117330	16	c.5445C>G	p.Tyr1815*	*De novo*	F	13.2
DL76010*	5	c.1810C>T	p.Arg604*		F	1.6
DL179067	22	c.6454C>T	p.Arg2152*	*De novo*	M	18
A1208	22	c.6454C>T	p.Arg2152*	*De novo*	F	3.5

Supplemental Table S5 – Pathogenic *NSD1* variants identified in individuals with Sotos syndrome. Pathogenic *NSD1* loss of function variants used in this study (taken from Supplemental Data 1 of Choufani et al. *Nature Communications* 2015, doi:10.1038/ncomms10207).

KMT2D pathogenic nonsense and frameshift variants

Sample ID	mutation DNA	mutation protein	Coding effect
KMT2D-1	c.15061C>T	p.Arg5021*	nonsense
KMT2D-2	c.16318delG	p.Glu5440Argfs*16	frameshift
KMT2D-3	c15030dupA	p.Glu5011Argfs*13	frameshift
KMT2D-4	c.8172_8173delC	p.Phe2724Glnfs*5	frameshift
KMT2D-5	c.6595delT	p.Tyr2199Ilefs*65	frameshift
KMT2D-6	c.14055-14056delCA	p.His4685Glnfs*4	frameshift
KMT2D-7	c.6295C>T	p.Arg2099*	nonsense
KMT2D-8	c.4135_4136delA	p.Met1379Valfs*52	frameshift
KMT2D-9	c.12592C>T	p.Arg4198*	nonsense
KMT2D-10	c.4135_4136delA	p.Met1379Valfs*52	frameshift
KMT2D-11	c.11710C>T	p.Gln3904*	nonsense

KMT2D missense variant

Sample ID	mutation DNA	mutation protein	Coding effect
KMT2D-12	c.15143G>A	p.Arg5048His	missense

Supplemental Table S6 – Pathogenic *KMT2D* variants identified in individuals with Kabuki syndrome. Pathogenic *KMT2D* variants used in this study (taken from Table S2 of Butcher et al. *American Journal of Human Genetics* 2017, dx.doi.org/10.1016/j.ajhg.2017.04.004).

		Nt	Tm, ºC	%GC	PCR Anneal ºC
cg02486855 F	TTGAATTTTTTTGGTGGGGAGAA	23	59.4	34.8	58
cg02486855 R	/5Biosg/CAAAACAACAACAAACCAATTAACAC	26	58.7	30.8	
cg02486855 Seq	TGGTGGGGAGAAAAG	15	48.3	53.3	
cg11106117 F	AATTTTTGAGGAAGAGTAGTAG	22	55.1	31.8	55
cg11106117 R	/5Biosg/AACCTCTAATACCCTATAATACTC	24	55.5	33.3	
cg11106117 Seq	TTTATTTAGAGTTTTGATTGT	21	41.2	19	
cg12902206 F	GGTGTGGTTTTGTGAGTTAAG	21	58.3	42.9	58
cg12902206 R	/5Biosg/CCACCCATCTTCCCTCCTATA	21	59	52.4	
cg12902206 Seq	TTTAGTGTTTTGATTAGTT	19	39.9	21.1	

Supplemental Table S7 – Primer sequences used for the pyrosequencing.
